# Disturbance sensitivity shapes patterns of tree species distribution in Afrotropical lowland rainforests more than climate or soil

**DOI:** 10.1002/ece3.11329

**Published:** 2024-05-01

**Authors:** Chase L. Núñez, James S. Clark, John R. Poulsen

**Affiliations:** ^1^ Department for the Ecology of Animal Societies Max Planck Institute of Animal Behavior Konstanz Germany; ^2^ Centre for the Advanced Study of Collective Behaviour University of Konstanz Konstanz Germany; ^3^ Department of Biology University of Konstanz Konstanz Germany; ^4^ University Program in Ecology Duke University Durham North Carolina USA; ^5^ Nicholas School of the Environment Duke University Durham North Carolina USA; ^6^ The Nature Conservancy Boulder Colorado USA

**Keywords:** Anthropocene, biodiversity, climate change, joint species distribution modeling, plant traits, tropical forest

## Abstract

Understanding how tropical forests respond to abiotic environmental changes is critical for preserving biodiversity, mitigating climate change, and maintaining ecosystem services in the coming century. To evaluate the relative roles of the abiotic environment and human disturbance on Central African tree community composition, we employ tree inventory data, remotely sensed climatic data, and soil nutrient data collected from 30 1‐ha plots distributed across a large‐scale observational experiment in forests that had been differently impacted by logging and hunting in northern Republic of Congo. We show that the composition of Afrotropical plant communities at this scale responds to human disturbance more than to climate, with particular sensitivities to hunting and distance to the nearest village (a proxy for other human activities, including tree‐cutting and gathering). These findings contrast neotropical predictions, highlighting the unique ecological, evolutionary, and anthropogenic history of Afrotropical forests.

## Graphical abstract

Posterior parameter estimates for the effect of environmental covariates on species counts and species correlation in their responses to environmental predictors show clustering into two general groups that represent a disturbance‐tolerant pioneer community and disturbance‐intolerant species.

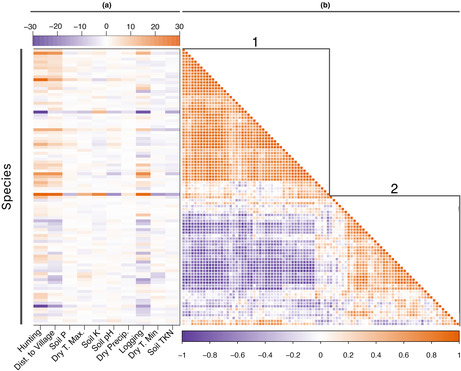

## 1 INTRODUCTION

Understanding how tropical forests respond to changes in human disturbance and in the abiotic environment is critical for preserving biodiversity, mitigating climate change, and maintaining ecosystem services in the coming century (Malhi et al., 2013a; Maslin et al., 2005; Núñez et al., 2022). The lowland rainforests of Central Africa, in particular, are expected to lose 41% of their forest cover to forest clearing by 2050 (Justice et al., 2001), largely due to the expansion of subsistence agriculture and logging (Tyukavina et al., 2018). This loss in forest cover contributes to further tree mortality from drought and fire through indirect microclimatic shifts (Condit et al., 1996, 2017; Martínez‐Vilalta & Lloret, 2016; Nepstad et al., 1999, 2004; Williamson et al., 2000). Even modest changes in available water may push this area into a climatic space untenable for rainforests (Guan et al., 2015; Malhi & Wright, 2004; Pan et al., 2011). Knowledge of how environmental disturbance has shaped current species and trait distributions could help more effectively allocate limited conservation resources to sensitive communities, yet our understanding of these relationships is poor.

Current understanding of environmental effects on tropical forest communities comes largely from studies that have focused primarily on the roles of drought and soil in neotropical forests. In contrast, the forests of Africa have fewer wet‐affiliated species than would be expected from climate–biodiversity relationships (Leal, 2009) and a high proportion of large trees that grow rapidly (Gond et al., 2013). These community‐level trait differences are hypothesized to have arisen from Africa's unique anthropogenic and evolutionary past (Haffer, 1969; Maley, 1996; Maslin et al., 2005; Oslisly et al., 2013; Willis et al., 2013), and may contribute to distinct responses to current environmental and anthropogenic pressures. Abnormally dry conditions during the last glacial maximum ~26,500 years ago affected all tropical forests, yet only Afrotropical forests were reduced to small patches of remnant forest (Haffer, 1969; Maley, 1996; Maslin et al., 2005, but see Pennington et al., 2000). These reductions are hypothesized to have selected for species adapted to water scarcity and the capacity to disperse from refugia to recolonize landscapes cleared by receding glaciers (Leal, 2009). Additionally, Holocene human disturbances associated with shifting cultivation have been suggested as a contributing factor to the observed low diversity (Parmentier et al., 2007) and high prevalence of light‐demanding species within the canopy (Almeida‐Rocha et al., 2020; Biwolé et al., 2015; Obiang et al., 2014; van Gemerden et al., 2003; Vleminckx et al., 2014; White & Oates, 1999). Indeed, paleoecological work shows evidence of Afrotropical forest communities' resilience to past climatic and anthropogenic disturbances. Although forest composition was not completely unchanged (Brncic et al., 2007), moist semi‐evergreen forest taxa have persisted through the last 3300 years with no sign of savannah expansion, despite evidence of anthropogenic activity and extensive periods of moisture limitation (Elenga et al., 2007).

These studies based on pollen records cannot be extrapolated to current and future forest composition due to the uneven representation of species in sedimentary deposits (Mander & Punyasena, 2018), so many researchers have turned to species distribution models (SDMs) that make use of recent presence‐only, presence–absence, or abundance data to anticipate changes in forest diversity (Elith & Leathwick, 2009). However, SDMs do not fully capture the degree to which species interactions mediate their response to the environment, contributing to a high level of uncertainty in predictions (Clark et al., 2017). This uncertainty is worsened by a reliance on parameters that have been fit at a simple level of aggregation (e.g., species‐scale) to predict responses at more complex levels of aggregation (e.g., community‐scale). The accumulation of error through aggregation is known as ‘Simpson's Paradox’ or the ‘ecological fallacy’ (Bickel et al., 1975; Clark et al., 2011). Finally, SDMs have traditionally been taxonomically limited because they are not able to cohesively integrate species data that are measured by different techniques and on different scales (Clark, 2016; Clark et al., 2017; Taylor‐Rodríguez et al., 2017). Improved parameter estimates with uncertainty that accounts for dependence between species can be obtained by using models that incorporate the joint community response (Clark et al., 2017; Núñez et al., 2022).

To evaluate the relative roles of the abiotic environment and disturbance on Central African tree community composition, we employ tree inventory data, remotely sensed historic climatic data, and soil nutrient data collected from 30 1‐ha plots distributed across a large‐scale observational study in lowland rainforests in the Republic of the Congo. Each of the 30 plots was differently impacted by logging and hunting. We then used plant trait data derived from the literature to explore the potential role of plant functional traits on current community composition. This is the first experiment to our knowledge that directly tests the relative effects of relative drivers of community structure and composition using an integrative data‐fusion approach. We predict that tree species in these communities will be more sensitive to human disturbance than climate and soil (Beirne, Miao, et al., 2019; Haffer, 1969; Maley, 1996; Maslin et al., 2005; Oslisly et al., 2013; Willis et al., 2013). Our null prediction is that these communities will mirror findings in neotropical forests and show the greatest relative sensitivity to dry season precipitation and temperatures (Engelbrecht et al., 2007), followed by soil nutrients (Clark et al., 1998; Harms et al., 2001; John et al., 2007; Phillips et al., 2003; Plotkin et al., 2000; Tuomisto et al., 1995) and human disturbance (Peres et al., 2010; Solar et al., 2016).

## 2 METHODS

### 2.1 Study area

**FIGURE 1:**
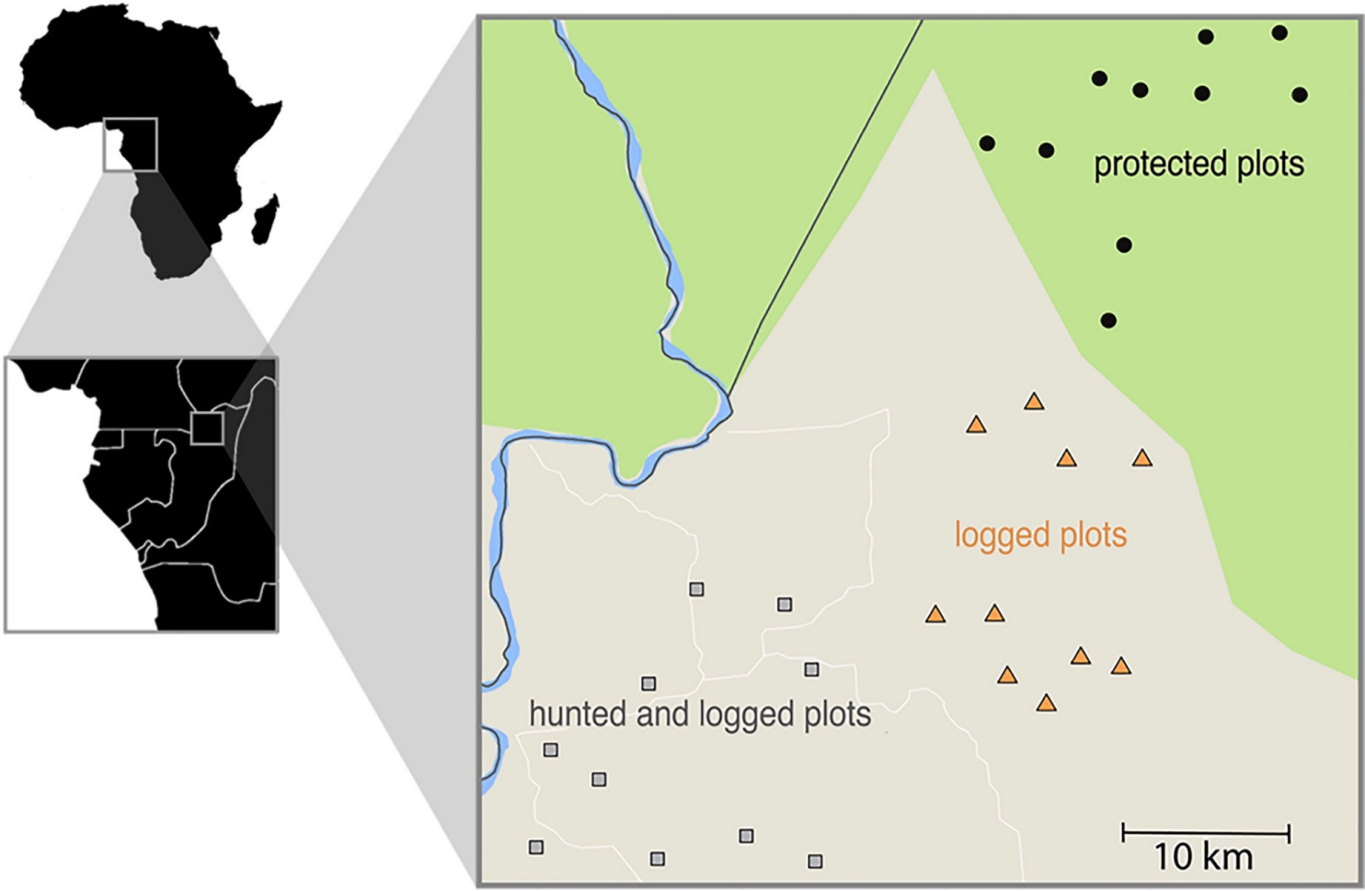
Location of 30 1‐hectare study plots in Central African Country of Republic of Congo (Brazzaville). Plots were situated in forest that were unlogged and unhunted (10 plots, circles), sites that were logged and unhunted (10 plots, triangles), and sites that were both logged and hunted (10 plots, squares). Protected plots fell within the border of Nouabalé‐Ndoki National Park whereas plots exposed to hunting and/or logging were located in the Kabo logging concession.

We conducted the study in Nouabale Ndoki National Park (NNNP; Longitude: 16°36′3″ East, Latitude: 2°32′22″ North; Area: 400,000 ha; Elevation: 442 m) and the Kabo logging concession (267,000 ha) in the Republic of the Congo (Figure 1). The diverse forests in this area are classified as lowland tropical forest where *Meliaceae*, *Euphorbiaceae*, and *Annonaceae* are the most represented families (CIB, 2006; Réjou‐Méchain et al., 2021). This area receives an average rainfall of about 1700 mm annually with seasonal peaks in May and October, and an intermediate dry season in December and January. The Kabo concession borders the NNNP to the south, and together they form a mosaic of logged and unlogged forest. About 20 years before the study began in 2005, the Kabo logging concession was selectively logged at low intensity (<2.5 stem ha^−1^) with four species making up 90% of the harvest volume: *Entandophragma cylindricum*, *E. utile*, *Triplochiton scleroxylon*, and *Milicia excelsa* (CIB, 2006). Approximately 3000 people inhabited the study site at the time of the study, most residing in the logging town of Kabo. Residents mainly hunt with shotguns, and occasionally with wire snares, for consumption and for local trade (Poulsen et al., 2009). A gradient of hunting intensity decreases with distance from Kabo (Poulsen et al., 2011), with some forest types being used more than others (Mockrin, 2008). This gradient is captured best in the Euclidian distance predictor variable ‘distance to nearest village’ because it shows the manifold types of human uses of forests that dissipate as human activities become less intense (Koerner et al., 2017).

### 2.2 Tree census data

We established 30 1‐ha tree plots situated in the forest that were categorized into three groups: 10 sites were unlogged and unhunted, 10 sites were logged and unhunted, and 10 sites were both logged and hunted. Using ArcView 3.2 and a 14‐class habitat map (Laporte et al., 2007), we randomly located plots within each disturbance regime in mixed lowland forest, with a buffer of at least 500 m to the nearest primary road and 100 m to the nearest water source. Within each plot, all trees greater than 10 cm in diameter at breast height (DBH) were tagged, measured, mapped, and identified to species (Wortley & Harris, 2014).

### 2.3 Plant species trait data

Life history traits for the tree species found in the 30 1‐ha plots were derived from the TRY global database of curated plant traits (Kattge et al., 2011). This included seed mass (Kühn et al., 2004), wood density (Zanne et al., 2009), and foliar traits (Kerkhoff et al., 2006; Kirkup et al., 2005; Kraft et al., 2008; Meng et al., 2015; Powers & Tiffin, 2010; Vergutz et al., 2012). Species‐level traits are often plastic responses to environmental conditions, and therefore repeated individual‐ and plot‐specific measures are preferable to determine not only mean values but also variance. However, in areas of high biodiversity where these data are not available or feasible to collect, careful use of existing trait data may help to illuminate broad‐scale patterns. Here, we explore the utility of archival trait data while acknowledging the limitations by using species‐specific trait values when available (<1% of species), and genus‐level averages when species‐level data were not found (~21%). If neither genus nor species‐level were available (~78%), we used family‐level averages. This method of phylogenetic trait interpolation allows us to use the best available information in an understudied area while not biasing the analysis. That is, species with less phylogenetically specific information will be supplied with values approaching the mean, and therefore have no predictive power on the response.

### 2.4 Soil data

We collected soil samples from the corners and center of each plot using a soil probe (2.85 × 83 cm) at 15 cm depth (c.f. Clark et al., 2012). Samples were weighed (wet mass), and then air‐dried and weighed again (dry mass). For analysis, the five samples from each plot were combined and homogenized before being sent to the Institute of Food and Agricultural Sciences Extension Soil Testing Laboratory at the University of Florida. Homogenized soil samples were analyzed for the percentage of sand, clay, and silt, as well as pH and nutrient availability (N, P, K, Al, Ca, Mg, Mn). Available cations and P were extracted using the Mehlich III solution (Tran & Simard, 1993). Elemental analysis for the cations and P was done on the extracts by using inductively coupled plasma spectroscopy. We extracted N as NH_4_ and NO_3_. Nitrogen was estimated calorimetrically using a Technicon II auto‐analyzer (SEAL Analytical, Mequon, Wisconsin, USA). The Kjeldahl method was used for the determination of total N (Hesse & Hesse, 1971). Soil pH was measured in an Adams‐Evans buffer solution made up of one volume of soil diluted in two volumes of water. Subsoil samples were analyzed for soil texture, using the hydrometer method (Sheldrick & Wang, 1993).

### 2.5 Climate data

Average recent historical precipitation, potential evapotranspiration, maximum temperature, and minimum temperature for each plot were derived from the NASA TerraClimate product (Abatzoglou et al., 2018) for 1985–2017 accessed using Google Earth Engine (Gorelick et al., 2017). To capture the contribution of seasonality, we amalgamated historical climate data before model selection. Climate values were averaged within the three recognized seasons in this region: long rains: (May–July), short rains (September–October), and dry season (November–April).

### 2.6 Generalized joint attribute model for species distributions

To accommodate the joint way that tree species in a community respond to environmental changes (Núñez et al., 2022), we employ a Generalized Joint Attribute Model (GJAM) to predict species abundance jointly, on the community scale (Clark et al., 2017). GJAM estimates can therefore be interpreted on the scale of the observations, accounting for sample effort. The model is based on a joint distribution [θ,X,Y] of parameters θ, predictors X, and species responses Y. Parameters (θ) in the model include matrices of coefficients relating X to Y (B) and the residual covariance matrix for all species pairs in Y (Σ). In effect, Σ represents the covariance between species beyond what has already been explained by the environmental covariates. It can include co‐dependence between species, unaccounted for environmental gradients, and other unexplained sources of error. The likelihood is: [Y_1_,…,Y_S_|θ,X], where subscripts refer to species 1 through S. Model fitting is done on the shared prediction/observation scale, based on the posterior distribution, [θ |X,Y] ∝ [Y1,…,YS|θ,X][θ], where the right‐hand side is the likelihood and the prior distribution, [θ], which is non‐informative. Sensitivity of the entire response matrix to environmental predictors can be obtained from the diagonal vector of the covariance matrix between predictors in X in terms of the responses they elicit from Y.

### 2.7 Generalized joint attribute model for traits

The incorporation of plant traits in GJAM is consistent with the structure of the model listed above, with community‐weighted mean (CWM) trait composition being predicted instead of species counts—i.e., the response is a trait by species matrix Y, which could be any trait or traits of interest. Use of CWM traits, or plot‐level trait averages weighted by the number of species in a plot (Muscarella & Uriarte, 2016), is a useful method of broadly characterizing communities, illuminating relationships between environmental gradients and patterns of life history adaptations and community assembly through environmental filtering (Cornwell & Ackerly, 2009; Sonnier et al., 2010; Wright et al., 2004). However, because trait data come in many forms (e.g., categorical classes, continuous measurements, discrete counts, etc.), GJAM leverages censoring with the effort for an observation to combine continuous and discrete variables with appropriate weight. In count data, effort is determined by the size of the sample plot and is comparable to the offset in GLMs. Full model specifications can be found in Clark et al. (2017). The suite of covariates (Appendix A1) included in the final model was selected by iteratively combining each covariate and comparing the Deviance Information Criterion (DIC) and Variance Inflation Factor (VIF) values for each model structure (including interactions) and selecting the optimal model that produced the most inference on the relationship between predictors and responses, while returning low DIC and VIF less than 3, indicating lack of collinearity among predictors. Model estimates were taken from 100,000 iterations, discarding the first 1000 iterations as pre‐convergence. We visually inspected trace plots to confirm convergence and adequate mixing (A2).

## 3 RESULTS

**FIGURE 2:**
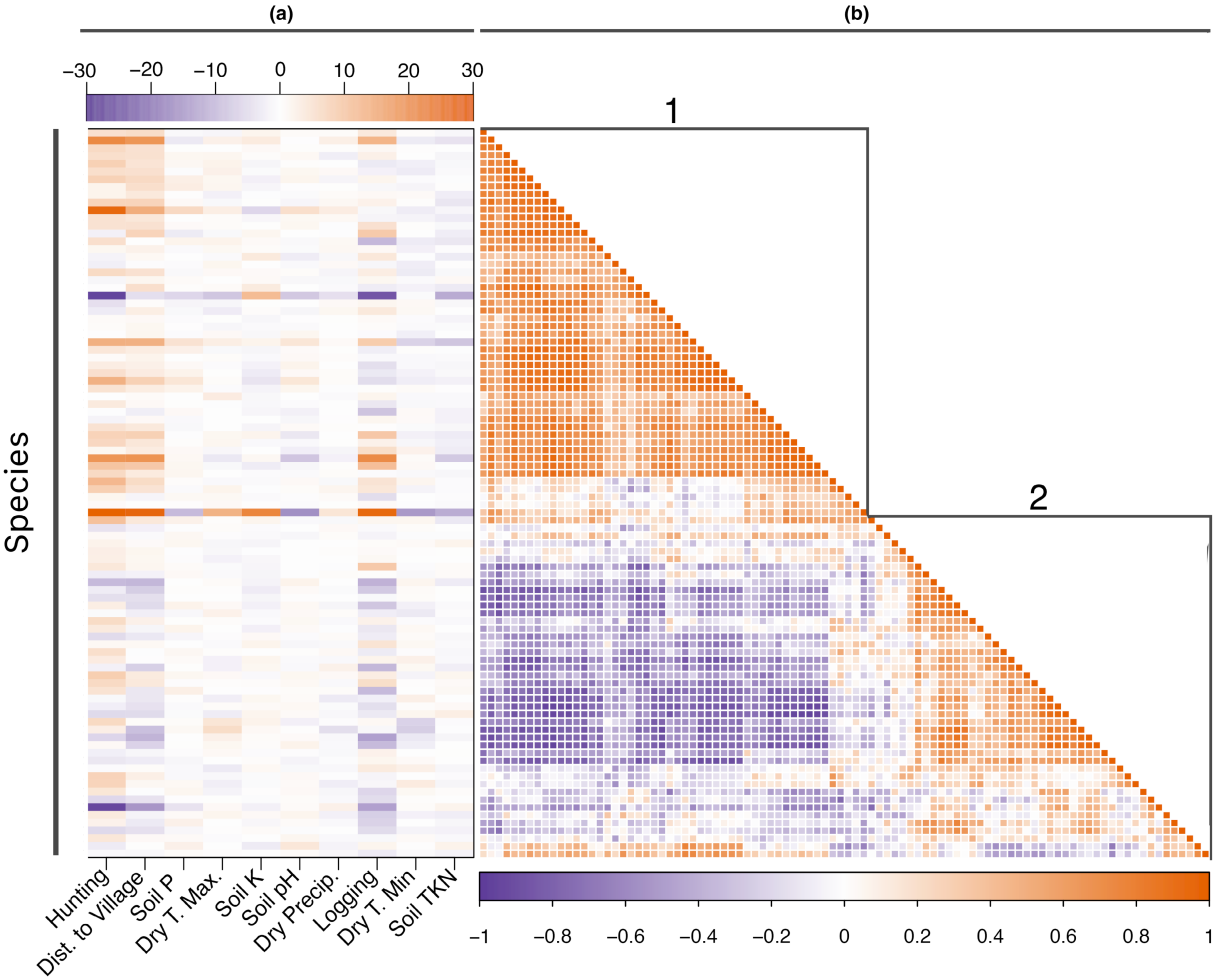
Posterior parameter estimates for the effect of environmental covariates on species counts (a) and species correlation in their responses to predictors in the model (b) show clustering into two general groups that represent a disturbance‐tolerant pioneer community (b1) and disturbance intolerant species (b2). Species names are listed in Appendices A9.

**FIGURE 3:**
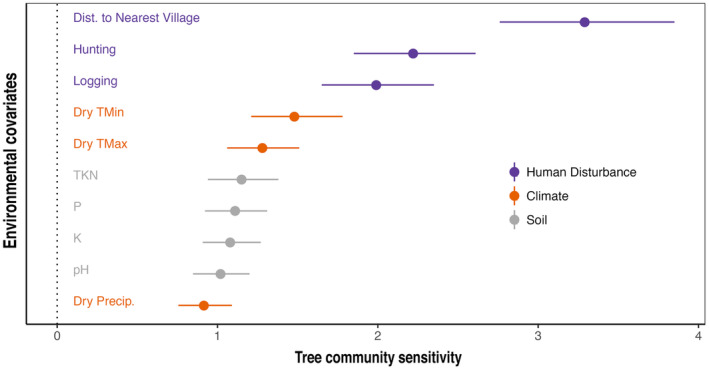
Sensitivity of community composition to environmental predictors and 95% credible interval (CI). Sensitivity is dimensionless and in the unit of the environmental covariate.

Responses to the environment, i.e., the effect of the environment on species abundance, varied among species (A3), but species occurrence was correlated within two clusters with opposing responses to predictor variables (Figure 2b). Group 1 (species positively associated with disturbance, Figure 2b1) was more likely to occur in plots disturbed by hunting, logging, or both, while group 2 (species negatively associated with disturbance, Figure 2b2) was most likely to occur in pristine plots undisturbed by hunting or logging. The varied responses of all species can be summarized by a comparison of community sensitivity to predictors across all species (Figure 3; A4), with greatest sensitivity (i.e., the relative increase in predictor to produce a one unit increase in species counts) to human disturbance (distance to nearest village 3.29 [2.76, 3.85], hunting 2.22 [1.85, 2.61], and logging 1.99 [1.65, 2.35]), followed by moderate sensitivity to dry season temperature (dry season minimum temperature 1.48 [1.21, 1.78], dry season maximum temperature 1.28 [1.06, 1.51]), and least sensitivity to dry season precipitation (0.92 [0.76, 1.09]) and soil composition Total Kjeldahl Nitrogen (TKN) (1.15 [0.94, 1.38]); P (1.11 [0.92, 1.31]); K (1.08 [0.91, 1.27]); pH (1.02 [0.85, 1.20]).

**FIGURE 4:**
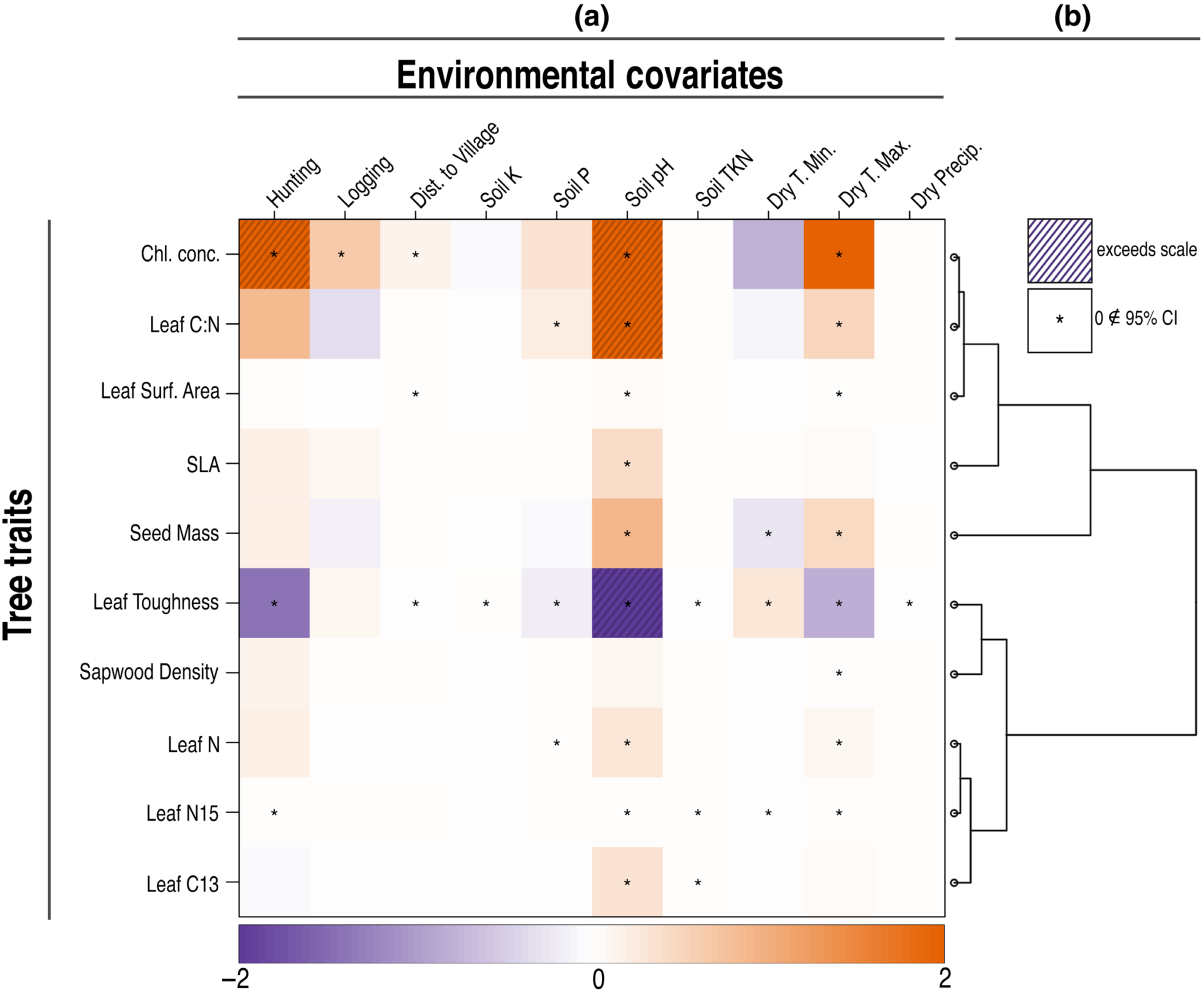
Standardized posterior parameter estimates for the effect of climatic, soil, and human disturbance predictors on community‐weighted trait values (a) and a dendrogram of correlation in trait response to predictors (b, Appendix A7 and A8).

Our trait analysis indicates that soil pH was informative of all traits except sapwood density. Soil nutrients were weakly informative of community‐weighted trait values: total nitrogen (TKN) was weakly related to leaf N (1.35 × 10^−06^ [2.70 × 10^−07^, 2.41 × 10^−06^]), and N‐15 (6.1 × 10^−04^ [9.62 × 10^−05^, 1.12 × 10^−03^]), as well as C‐13 (−1.27 × 10^−03^ [−1.74 × 10^−03^, −8.04 × 10^−04^]). Soil P was moderately informative of the distribution of species with high leaf C:N ratios (0.20 [0.02, 0.38]), C13 (−0.18 [−0.24, −0.12]), and leaf surface area (0.04 [0.00, 0.07]). Soil K was weakly informative of species with greater leaf C13 (0.02 [0.01, 0.03]) (Figure 4a; A5).

**FIGURE 5:**
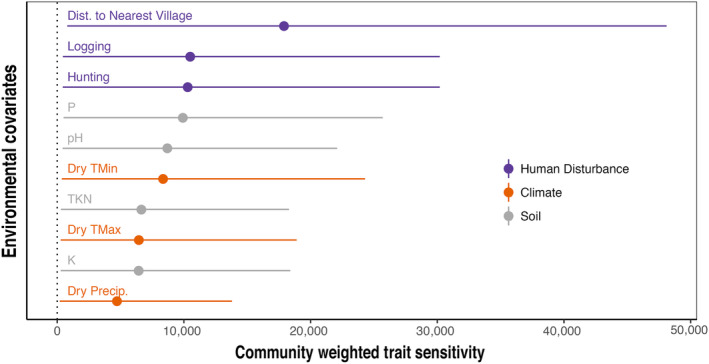
Sensitivity of community‐weighted trait values to environmental covariates and 95% credible interval (CI). Sensitivity is dimensionless and in the unit of the environmental covariate.

There was a strong association between dry‐season climate and community‐weighted leaf traits. Maximum temperature was related to chlorophyll concentration (2.00 [1.37, 2.65]), carbon to nitrogen ratio (0.52 [0.25, 0.78]), surface area (0.11 [0.06, 0.16]), toughness (0.04 [0.00, 0.07]), sapwood density (0.02 [0.01, 0.03]), C13 (−0.84 [−0.93, −0.75]), and SLA (0.48 [0.28, 0.68]) (Figure 5; A6).

Disturbance was strongly associated with chlorophyll concentrations (hunting (3.75 [0.85, 6.65]), distance to nearest village (0.16 [0.05, 0.28])), leaf C13 (hunting (−1.42 [−1.82, −1.02]), and distance to nearest village (−0.03 [−0.05, −0.02])).

Community weighted traits covaried in two groups: one encompassing the similar responses of chlorophyll concentration, leaf C:N, leaf surface area, SLA, and seed mass to environmental predictors, and the other encompassing the similar responses of leaf toughness, sapwood density, and leaf N, N15, and C13 (Figure 4b). Community‐weighted traits were most sensitive to human disturbance (distance to nearest village 17,900 [793, 48,100], hunting 10,300 [437, 30,200], and logging 10,500 [447, 30,200]); followed by moderate sensitivity to soil composition TKN (6650 [289, 18,300]), P (9920 [499, 25,700]), K (6430 [268, 18,400]), pH (8700 [419, 22,100]), and dry season climate (dry season minimum temperature 8370 [360, 24,300], dry season maximum temperature 6450 [274, 18,900], and dry season precipitation 4720 [198, 13,800]) (Figure 5).

## 4 DISCUSSION

We find that Afrotropical plant communities are more closely tied to human disturbance than to climate or soil. This result contrasts with neotropical studies that show strong sensitivity to dry season precipitation and temperatures (Engelbrecht et al., 2007), and soil nutrients (Clark et al., 1998; Fayolle et al., 2014; Harms et al., 2001; John et al., 2007; Phillips et al., 2003; Plotkin et al., 2000; Tuomisto et al., 1995). We show that responses to the abiotic environment varied among species (Figure 2a), but species' distribution was organized into two groups with opposing responses to predictor variables (Figure 2b), particularly hunting and distance to the village (an inclusive proxy for other human activity) that disproportionately affect species composition in these plots (Figure 3). The ultimate effect of a predictor variable will always depend on the spatiotemporal range in the data—a weakly informative human effect may have a strong effect where climate variation is negligible. However, here we report there are strong human effects across a wide climatic gradient, lending strength to our finding. Human activity has well‐known negative effects on Central African animal communities (Beirne, Nuñez, et al., 2019; Koerner et al., 2017; Núñez, Froese, et al., 2019; Poulsen et al., 2011, 2017, 2021), and our results suggest that these disturbance gradients are also related to tree community composition, supporting past work suggesting indirect stress on plants and alterations in plant–animal interactions can modify plant community composition (Núñez, Clark, et al., 2019; Poulsen et al., 2013).

Our trait (Figure 4a) and sensitivity analyses (Figure 5) demonstrate that this grouped response of species to human disturbance is likely being driven by traits associated with two distinct successional communities. Despite having limited species‐specific trait information, human disturbance predictors were still strongly related to a subset of foliar traits common to disturbance‐related pioneer species—higher chlorophyll concentrations and leaf surface area, with lower leaf toughness. The broad overlap in sensitivity of community‐weighted traits to environmental predictors could be due in part to coarse trait data (see below), but could also indicate that these communities are not being assembled through systematic trait‐filtering, but rather a species‐specific response to both measured and unmeasured variables in ways that are not readily decomposed into traits (Clark, 2016).

Contrary to past studies finding a strong effect of soil composition on species composition (Amazonia: Tuomisto et al., 1995; Costa Rica: Clark et al., 1998; Malaysia: Plotkin et al., 2000; Panama: Harms et al., 2001; South‐West Amazonia, Phillips et al., 2003; Colombia, Ecuador, and Panama: John et al., 2007, African Tropical Forests from Senegal to Mozambique: Fayolle et al., 2014), our results indicate that soil heterogeneity had weaker effects on tree communities than other variables (Figure 3). Soil characteristics were more strongly related to community‐weighted leaf traits in accordance with ecophysiological theory (Mayor et al., 2014; Ordoñez et al., 2009; Vitousek, 1984), including a particularly strong relationship between soil pH and all plant traits except sapwood density (Figure 4a). This is likely because soil pH indirectly affects the uptake and availability of several plant nutrients. Ca, Mg, K, and P are less available in low pH soils, whereas Al, Cu, Mn, and Zn cations become more available in low pH soils (Daniels, 2016; John et al., 2007). Nevertheless, it is important to consider tropical forest soil dynamics when interpreting these results. Tropical soils are notoriously nutrient poor as a result of age, leaching, and most nutrient matter being held in the aboveground biomass (Jordan & Herrera, 1981); however, it is likely that the observed soil nutrient composition at the time of study does not accurately represent the nutrient availability when the current adult trees were dispersed as seeds and recruited into the canopy (Muledi et al., 2020).

Climate had moderate effects on community composition as species abundances were most sensitive to dry season temperature and to some degree sensitive to dry season precipitation (Figure 3). There was also a strong relationship between dry season climate and community‐weighted leaf traits (Figure 4a), likely due to the role those traits have in moderating evapotranspiration and maximizing photosynthesis. These effects, although slight in comparison to human disturbance, could have large effects on forest composition in light of the rapid increases in temperature predicted for this area (Leal, 2009; Malhi et al., 2013b)—up to a 6°C increase by the end of the century in the high greenhouse gas emission scenario (Climate Service Center [CSC], 2011).

Care needs to be taken when interpreting results from tree plots. Although individual species locations and identities in our dataset are quite refined, environmental predictor data (minimum/maximum temperature, precipitation) are spatially coarse. The spatial extent of our study design covers ~4000 km^2^ and three disturbance regimes, but there is still relatively little variation in climate at this scale, potentially reducing the predictive power of our models. Additionally, the strong effects of human disturbance could be partially due to our study design, which was necessarily pseudoreplicated, i.e., study plots affected by the same disturbance type were geographically grouped together because human disturbance was also geographically concentrated. This was a direct result of the spatial pattern of hunting and logging around the village of Kabo (Poulsen et al., 2011), and means that other, unmeasured environmental gradients could have influenced our results. Future work should be conducted at a larger spatial extent (e.g., nationally with independent replicates for each disturbance regime) to see if the effects of disturbance hold up at a larger spatial scale. Additionally, interpolated trait data require careful post hoc interpretation. Trait data do not exist for most tropical species and therefore values used are community averages for each plot calculated from the low proportion of species for which data were available. Results showing similarities in response are therefore related to both trait values as well as phylogenetic clustering. To model future responses of tree species and communities to changing climate, we need higher resolution species trait and local weather data, both lacking in developing tropical areas. Effort and investment should be committed to collecting trait data for tropical tree species for upload onto open databases like TRY (Kattge et al., 2011, 2020), as well as for increasing the number of local weather stations across central Africa.

Our findings represent the most thorough study so far linking the joint responses of Afrotropical tree species distribution patterns with species' environmental responses. The response of tropical plant species to environmental gradients like soil nutrients, light, and water have been experimentally measured in the past (Clark et al., 2016; Ewel & Mazzarino, 2008; Hill & Hamer, 2004; Martínez‐Vilalta & Lloret, 2016; Palmiotto et al., 2004), but how these responses control whole communities has been less well understood. This is the first study to our knowledge to jointly model the response of Afrotropical forest communities to soil, climate, and disturbance. Our results emphasize the sensitivity of tropical forests to both human activity and climate, with human disturbance having a direct role in determining species distributions in concert with local and regional water availability. Thus, the future expansion of humans into central African forests coupled with dramatic increases in temperature expected by the end of the century will have direct consequences for species ranges, tropical forest community composition and ecosystem function. Conservation of tree communities will involve mitigating disturbances of hunting and logging in the short term and climate change in the long term. Current vegetation–climate models, particularly for tropical regions, suffer from a lack of ecological data and mechanistic understanding of the factors shaping current species distributions. The knowledge that dry season temperature, together with the sensitivity of species to human disturbance, is influencing species distribution patterns in tropical forests will help to improve the accuracy and specificity of predictions of vegetation shifts under global change scenarios.

## AUTHOR CONTRIBUTIONS

**Chase L. Núñez:** Conceptualization (lead); data curation (lead); formal analysis (lead); funding acquisition (lead); investigation (lead); methodology (lead); project administration (lead); resources (lead); software (equal); validation (lead); visualization (lead); writing – original draft (lead); writing – review and editing (lead). **James S. Clark:** Software (equal); supervision (supporting); writing – review and editing (supporting). **John R. Poulsen:** Resources (equal); supervision (supporting); writing – review and editing (supporting).

## FUNDING INFORMATION

CLN received funding from Deutsche Forschungsgemeinschaft EXC‐2035/1‐390681379, and National Science Foundation GRF‐1106401.

## CONFLICT OF INTEREST STATEMENT

The authors declare that the research was conducted in the absence of any commercial or financial relationships that could be construed as a potential conflict of interest.

## Supporting information


Appendix S1

## Data Availability

The data are subject to third‐party restrictions. Requests to access these datasets should be directed to JP, john.poulsen@duke.edu.
