# Correction to Disturbance sensitivity shapes patterns of tree species distribution in Afrotropical lowland rainforests more than climate or soil

**DOI:** 10.1002/ece3.70083

**Published:** 2024-07-22

Núñez, C. L., Clark, J. S., & Poulsen, J. R. (2024). Disturbance sensitivity shapes patterns of tree species distribution in Afrotropical lowland rainforests more than climate or soil. *Ecology and Evolution*, 14, e11329. https://doi.org/10.1002/ece3.11329

In the funding information section, the institutional granting code is incorrect.

Where it currently reads: “CLN received funding from Deutsche Forschungsgemeinschaft EXC‐2035/1‐390681379, and National Science Foundation GRF‐1106401.”, it should read: “CLN received funding from Deutsche Forschungsgemeinschaft (DFG, German Research Foundation) under Germany's Excellence Strategy – EXC 2117 – 422037984, and National Science Foundation GRF‐1106401.” We apologize for this error.

