# Supplementary material for: Disturbance sensitivity shapes patterns of tree species distribution in Afrotropical lowland rainforests more than climate or soil
Source: Ecol Evol. 2024 May 1;14(5):e11329. doi: 10.1002/ece3.11329 (PMC11063613; doi:10.1002/ece3.11329)

# Appendix A

# Disturbance Sensitivity Shapes Patterns of Tree Species Distribution in Afrotropical Lowland Rainforests More Than Climate or Soil

## A1: Comparison of scaled covariates across 30 study plots shows greatest climatic variation across plots in dry season and heterogeneous plot-level soil characteristics. S. and L. rains are short and long rainy season respectively.
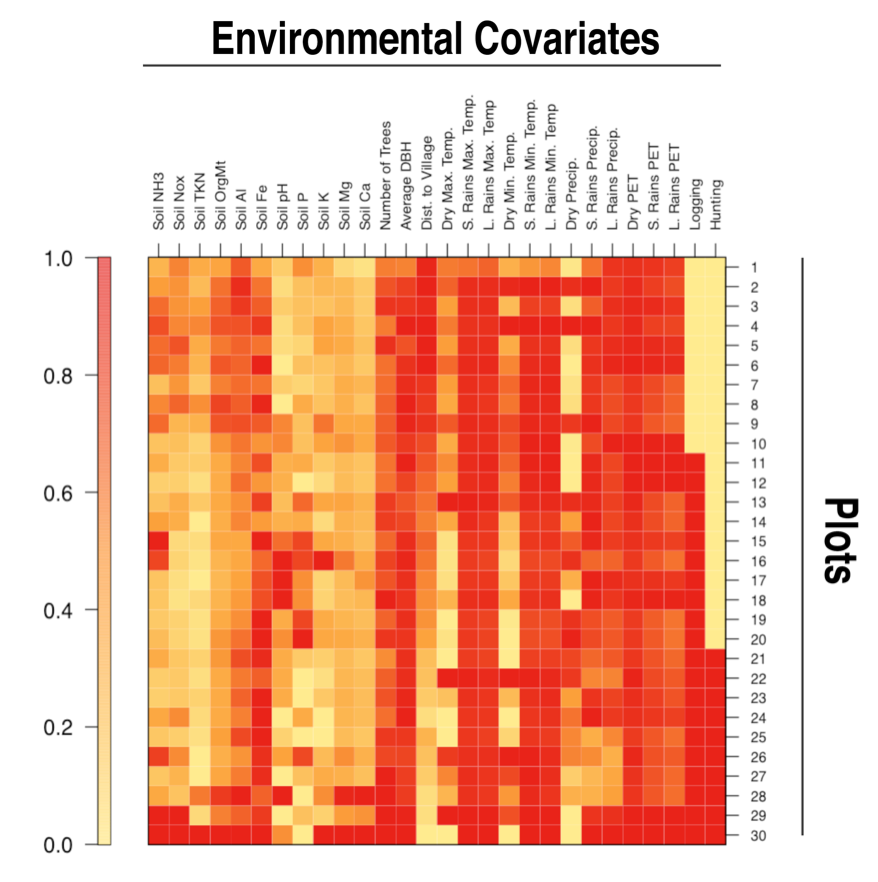


## A2: Trace of posterior chains for each environmental covariate shows convergence of parameter estimates.


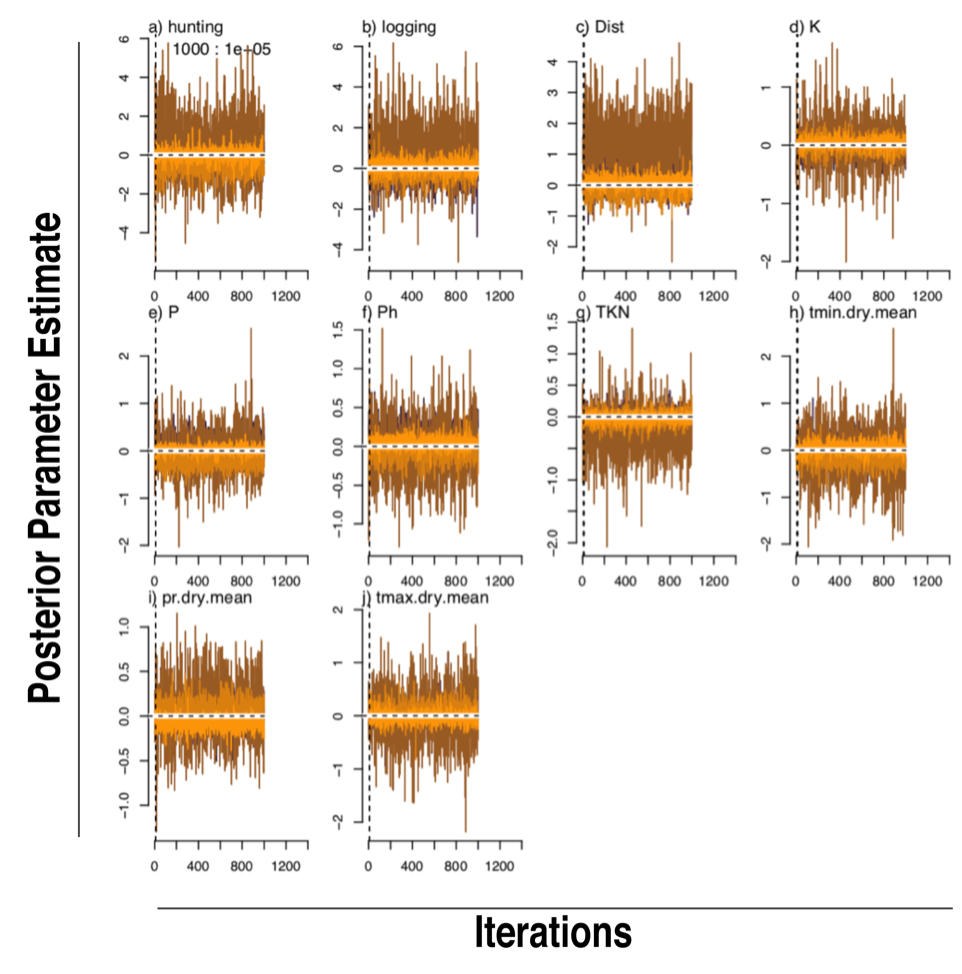


## A3: Species-specific posterior parameter estimates for the effect of each environmental covariate on species counts.

| Posterior Parameter Estimates for Species | | | | | | | | | | |
| --- | --- | --- | --- | --- | --- | --- | --- | --- | --- | --- |
|  |  |  |  | Estimate |  | 2.50% |  | 97.50% |  | 0 ∉ 95% CI |
| Afrostyrax lepidophyllus | | | | |  |  |  |  |  |  |
|  |  | intercept |  | 0.77 |  | -0.04 |  | 1.62 |  |  |
|  |  | hunting1 |  | 6.63 |  | -3.85 |  | 17.20 |  |  |
|  |  | logging1 |  | -3.41 |  | -13.00 |  | 6.06 |  |  |
|  |  | Dist |  | 0.24 |  | -0.16 |  | 0.64 |  |  |
|  |  | K |  | -0.18 |  | -0.35 |  | -0.02 |  | * |
|  |  | P |  | 1.27 |  | -0.35 |  | 2.93 |  |  |
|  |  | Ph |  | 5.17 |  | -2.42 |  | 13.10 |  |  |
|  |  | TKN |  | 0.00 |  | -0.02 |  | 0.01 |  |  |
|  |  | tmin.dry.mean | | -1.75 |  | -4.79 |  | 1.15 |  |  |
|  |  | pr.dry.mean |  | 0.02 |  | -0.04 |  | 0.08 |  |  |
|  |  | tmax.dry.mean | | 0.68 |  | -1.52 |  | 2.93 |  |  |
| Afzelia bipindensis | | |  |  |  |  |  |  |  |  |
|  |  | intercept |  | 0.44 |  | -0.34 |  | 1.22 |  |  |
|  |  | hunting1 |  | 3.35 |  | -6.08 |  | 12.80 |  |  |
|  |  | logging1 |  | -0.92 |  | -9.81 |  | 7.88 |  |  |
|  |  | Dist |  | 0.04 |  | -0.33 |  | 0.41 |  |  |
|  |  | K |  | 0.01 |  | -0.14 |  | 0.16 |  |  |
|  |  | P |  | 0.32 |  | -1.11 |  | 1.73 |  |  |
|  |  | Ph |  | 4.09 |  | -2.68 |  | 10.90 |  |  |
|  |  | TKN |  | 0.00 |  | -0.02 |  | 0.01 |  |  |
|  |  | tmin.dry.mean | | 0.96 |  | -1.86 |  | 3.78 |  |  |
|  |  | pr.dry.mean |  | -0.02 |  | -0.08 |  | 0.03 |  |  |
|  |  | tmax.dry.mean | | -1.14 |  | -3.32 |  | 1.02 |  |  |
| Albizia gummifera | | |  |  |  |  |  |  |  |  |
|  |  | intercept |  | 0.44 |  | -0.34 |  | 1.25 |  |  |
|  |  | hunting1 |  | 6.83 |  | -3.67 |  | 17.60 |  |  |
|  |  | logging1 |  | 3.33 |  | -8.70 |  | 15.80 |  |  |
|  |  | Dist |  | 0.19 |  | -0.25 |  | 0.66 |  |  |
|  |  | K |  | -0.02 |  | -0.17 |  | 0.12 |  |  |
|  |  | P |  | 0.47 |  | -1.09 |  | 2.08 |  |  |
|  |  | Ph |  | 1.75 |  | -5.61 |  | 9.05 |  |  |
|  |  | TKN |  | 0.01 |  | -0.01 |  | 0.02 |  |  |
|  |  | tmin.dry.mean | | -0.91 |  | -3.88 |  | 1.99 |  |  |
|  |  | pr.dry.mean |  | 0.02 |  | -0.04 |  | 0.08 |  |  |
|  |  | tmax.dry.mean | | -0.31 |  | -2.49 |  | 1.88 |  |  |
| Amphimas pterocarpoides | | | | |  |  |  |  |  |  |
|  |  | intercept |  | 0.24 |  | -0.48 |  | 0.96 |  |  |
|  |  | hunting1 |  | 4.88 |  | -4.23 |  | 14.20 |  |  |
|  |  | logging1 |  | 6.27 |  | -3.10 |  | 15.90 |  |  |
|  |  | Dist |  | 0.23 |  | -0.16 |  | 0.63 |  |  |
|  |  | K |  | 0.05 |  | -0.09 |  | 0.20 |  |  |
|  |  | P |  | -0.41 |  | -1.91 |  | 1.06 |  |  |
|  |  | Ph |  | 0.94 |  | -5.55 |  | 7.45 |  |  |
|  |  | TKN |  | 0.00 |  | -0.01 |  | 0.01 |  |  |
|  |  | tmin.dry.mean | | 0.61 |  | -2.13 |  | 3.36 |  |  |
|  |  | pr.dry.mean |  | -0.03 |  | -0.09 |  | 0.03 |  |  |
|  |  | tmax.dry.mean | | -0.94 |  | -3.12 |  | 1.17 |  |  |
| Angylocalyx pynaertii | | | |  |  |  |  |  |  |  |
|  |  | intercept |  | 0.16 |  | -0.58 |  | 0.89 |  |  |
|  |  | hunting1 |  | 2.68 |  | -6.53 |  | 11.80 |  |  |
|  |  | logging1 |  | 5.76 |  | -2.92 |  | 14.50 |  |  |
|  |  | Dist |  | 0.30 |  | -0.07 |  | 0.66 |  |  |
|  |  | K |  | 0.04 |  | -0.09 |  | 0.18 |  |  |
|  |  | P |  | 0.11 |  | -1.24 |  | 1.46 |  |  |
|  |  | Ph |  | 1.21 |  | -5.35 |  | 7.75 |  |  |
|  |  | TKN |  | -0.01 |  | -0.02 |  | 0.00 |  |  |
|  |  | tmin.dry.mean | | 0.78 |  | -1.86 |  | 3.43 |  |  |
|  |  | pr.dry.mean |  | -0.01 |  | -0.07 |  | 0.04 |  |  |
|  |  | tmax.dry.mean | | -0.97 |  | -2.95 |  | 1.01 |  |  |
| Anonidium mannii | | |  |  |  |  |  |  |  |  |
|  |  | intercept |  | 0.05 |  | -0.65 |  | 0.77 |  |  |
|  |  | hunting1 |  | 3.05 |  | -5.70 |  | 11.70 |  |  |
|  |  | logging1 |  | -1.98 |  | -10.10 |  | 6.10 |  |  |
|  |  | Dist |  | 0.00 |  | -0.35 |  | 0.35 |  |  |
|  |  | K |  | -0.07 |  | -0.20 |  | 0.07 |  |  |
|  |  | P |  | 0.95 |  | -0.36 |  | 2.27 |  |  |
|  |  | Ph |  | -1.71 |  | -8.07 |  | 4.64 |  |  |
|  |  | TKN |  | 0.00 |  | -0.01 |  | 0.01 |  |  |
|  |  | tmin.dry.mean | | -0.77 |  | -3.31 |  | 1.75 |  |  |
|  |  | pr.dry.mean |  | 0.02 |  | -0.03 |  | 0.07 |  |  |
|  |  | tmax.dry.mean | | 0.94 |  | -1.00 |  | 2.88 |  |  |
| Anthonotha macrophylla | | | | |  |  |  |  |  |  |
|  |  | intercept |  | -0.28 |  | -1.22 |  | 0.64 |  |  |
|  |  | hunting1 |  | 6.33 |  | -5.41 |  | 18.20 |  |  |
|  |  | logging1 |  | 1.26 |  | -13.50 |  | 16.30 |  |  |
|  |  | Dist |  | 0.02 |  | -0.50 |  | 0.56 |  |  |
|  |  | K |  | 0.14 |  | -0.03 |  | 0.33 |  |  |
|  |  | P |  | 0.19 |  | -1.64 |  | 2.01 |  |  |
|  |  | Ph |  | -9.32 |  | -19.40 |  | 0.19 |  |  |
|  |  | TKN |  | 0.00 |  | -0.02 |  | 0.01 |  |  |
|  |  | tmin.dry.mean | | -5.22 |  | -9.44 |  | -1.17 |  | * |
|  |  | pr.dry.mean |  | -0.01 |  | -0.10 |  | 0.08 |  |  |
|  |  | tmax.dry.mean | | 4.11 |  | 1.12 |  | 7.19 |  | * |
| Antides malaciniatum | | | |  |  |  |  |  |  |  |
|  |  | intercept |  | 0.01 |  | -0.79 |  | 0.80 |  |  |
|  |  | hunting1 |  | -3.66 |  | -14.00 |  | 6.38 |  |  |
|  |  | logging1 |  | -4.76 |  | -13.40 |  | 3.72 |  |  |
|  |  | Dist |  | -0.31 |  | -0.70 |  | 0.07 |  |  |
|  |  | K |  | 0.07 |  | -0.07 |  | 0.22 |  |  |
|  |  | P |  | -0.50 |  | -1.95 |  | 0.96 |  |  |
|  |  | Ph |  | 2.25 |  | -4.73 |  | 9.18 |  |  |
|  |  | TKN |  | 0.00 |  | -0.01 |  | 0.01 |  |  |
|  |  | tmin.dry.mean | | 0.60 |  | -2.17 |  | 3.37 |  |  |
|  |  | pr.dry.mean |  | -0.02 |  | -0.08 |  | 0.04 |  |  |
|  |  | tmax.dry.mean | | -0.36 |  | -2.42 |  | 1.73 |  |  |
| Barteria fistulosa | | |  |  |  |  |  |  |  |  |
|  |  | intercept |  | 0.32 |  | -0.52 |  | 1.16 |  |  |
|  |  | hunting1 |  | 4.47 |  | -5.88 |  | 15.30 |  |  |
|  |  | logging1 |  | -10.40 |  | -22.60 |  | 0.64 |  |  |
|  |  | Dist |  | -0.25 |  | -0.64 |  | 0.13 |  |  |
|  |  | K |  | 0.00 |  | -0.17 |  | 0.17 |  |  |
|  |  | P |  | 1.38 |  | -0.34 |  | 3.17 |  |  |
|  |  | Ph |  | 0.88 |  | -6.70 |  | 8.21 |  |  |
|  |  | TKN |  | 0.01 |  | -0.01 |  | 0.02 |  |  |
|  |  | tmin.dry.mean | | 0.07 |  | -3.06 |  | 3.13 |  |  |
|  |  | pr.dry.mean |  | 0.01 |  | -0.05 |  | 0.08 |  |  |
|  |  | tmax.dry.mean | | -0.34 |  | -2.64 |  | 1.98 |  |  |
| Beilschmiedia sp. | | |  |  |  |  |  |  |  |  |
|  |  | intercept |  | 0.50 |  | -0.48 |  | 1.50 |  |  |
|  |  | hunting1 |  | 2.06 |  | -9.71 |  | 13.70 |  |  |
|  |  | logging1 |  | -7.17 |  | -17.70 |  | 3.04 |  |  |
|  |  | Dist |  | -0.15 |  | -0.62 |  | 0.31 |  |  |
|  |  | K |  | 0.09 |  | -0.07 |  | 0.25 |  |  |
|  |  | P |  | 0.31 |  | -1.31 |  | 1.95 |  |  |
|  |  | Ph |  | 4.21 |  | -3.71 |  | 12.60 |  |  |
|  |  | TKN |  | -0.01 |  | -0.03 |  | 0.00 |  | * |
|  |  | tmin.dry.mean | | -1.23 |  | -4.76 |  | 2.36 |  |  |
|  |  | pr.dry.mean |  | -0.06 |  | -0.13 |  | 0.01 |  |  |
|  |  | tmax.dry.mean | | 0.80 |  | -1.97 |  | 3.51 |  |  |
| Blighia welwitschii | | |  |  |  |  |  |  |  |  |
|  |  | intercept |  | 0.38 |  | -0.37 |  | 1.15 |  |  |
|  |  | hunting1 |  | 5.66 |  | -3.84 |  | 15.20 |  |  |
|  |  | logging1 |  | 4.15 |  | -5.07 |  | 13.40 |  |  |
|  |  | Dist |  | 0.41 |  | 0.00 |  | 0.83 |  | * |
|  |  | K |  | 0.11 |  | -0.05 |  | 0.28 |  |  |
|  |  | P |  | -0.49 |  | -1.94 |  | 0.95 |  |  |
|  |  | Ph |  | 2.38 |  | -4.20 |  | 9.03 |  |  |
|  |  | TKN |  | -0.01 |  | -0.02 |  | 0.01 |  |  |
|  |  | tmin.dry.mean | | 0.35 |  | -2.37 |  | 3.09 |  |  |
|  |  | pr.dry.mean |  | 0.01 |  | -0.04 |  | 0.07 |  |  |
|  |  | tmax.dry.mean | | -1.42 |  | -3.57 |  | 0.68 |  |  |
| Camptostylus mannii | | | |  |  |  |  |  |  |  |
|  |  | intercept |  | 0.21 |  | -0.53 |  | 0.95 |  |  |
|  |  | hunting1 |  | 1.80 |  | -7.82 |  | 11.20 |  |  |
|  |  | logging1 |  | 0.72 |  | -7.60 |  | 9.13 |  |  |
|  |  | Dist |  | 0.09 |  | -0.29 |  | 0.48 |  |  |
|  |  | K |  | -0.09 |  | -0.25 |  | 0.05 |  |  |
|  |  | P |  | -0.11 |  | -1.51 |  | 1.30 |  |  |
|  |  | Ph |  | 1.26 |  | -5.38 |  | 7.84 |  |  |
|  |  | TKN |  | -0.01 |  | -0.02 |  | 0.00 |  |  |
|  |  | tmin.dry.mean | | -0.70 |  | -3.27 |  | 1.87 |  |  |
|  |  | pr.dry.mean |  | 0.01 |  | -0.05 |  | 0.06 |  |  |
|  |  | tmax.dry.mean | | 0.82 |  | -1.15 |  | 2.81 |  |  |
| Carapa procera | | |  |  |  |  |  |  |  |  |
|  |  | intercept |  | 0.08 |  | -0.68 |  | 0.86 |  |  |
|  |  | hunting1 |  | 4.48 |  | -4.96 |  | 14.20 |  |  |
|  |  | logging1 |  | 1.89 |  | -6.71 |  | 10.80 |  |  |
|  |  | Dist |  | 0.01 |  | -0.37 |  | 0.40 |  |  |
|  |  | K |  | 0.09 |  | -0.05 |  | 0.25 |  |  |
|  |  | P |  | 0.45 |  | -0.96 |  | 1.88 |  |  |
|  |  | Ph |  | -0.84 |  | -7.78 |  | 6.13 |  |  |
|  |  | TKN |  | 0.00 |  | -0.01 |  | 0.01 |  |  |
|  |  | tmin.dry.mean | | 1.81 |  | -0.92 |  | 4.54 |  |  |
|  |  | pr.dry.mean |  | -0.02 |  | -0.08 |  | 0.04 |  |  |
|  |  | tmax.dry.mean | | -1.29 |  | -3.39 |  | 0.80 |  |  |
| Celtis adolfi friderici | | | |  |  |  |  |  |  |  |
|  |  | intercept |  | 0.34 |  | -0.39 |  | 1.06 |  |  |
|  |  | hunting1 |  | -1.87 |  | -10.80 |  | 7.05 |  |  |
|  |  | logging1 |  | -8.20 |  | -16.50 |  | 0.00 |  |  |
|  |  | Dist |  | -0.47 |  | -0.83 |  | -0.12 |  | * |
|  |  | K |  | -0.18 |  | -0.33 |  | -0.03 |  | * |
|  |  | P |  | 0.28 |  | -1.06 |  | 1.61 |  |  |
|  |  | Ph |  | 4.67 |  | -1.84 |  | 11.20 |  |  |
|  |  | TKN |  | 0.00 |  | -0.01 |  | 0.01 |  |  |
|  |  | tmin.dry.mean | | 0.31 |  | -2.34 |  | 2.93 |  |  |
|  |  | pr.dry.mean |  | -0.01 |  | -0.07 |  | 0.04 |  |  |
|  |  | tmax.dry.mean | | 0.71 |  | -1.30 |  | 2.77 |  |  |
| Celtis mildbraedii | | |  |  |  |  |  |  |  |  |
|  |  | intercept |  | 1.46 |  | 0.18 |  | 2.80 |  | * |
|  |  | hunting1 |  | 11.40 |  | -3.01 |  | 26.30 |  |  |
|  |  | logging1 |  | 9.80 |  | -3.03 |  | 23.90 |  |  |
|  |  | Dist |  | 0.88 |  | 0.30 |  | 1.50 |  | * |
|  |  | K |  | 0.17 |  | -0.14 |  | 0.48 |  |  |
|  |  | P |  | 2.20 |  | -0.60 |  | 5.13 |  |  |
|  |  | Ph |  | 11.40 |  | -2.18 |  | 25.10 |  |  |
|  |  | TKN |  | -0.05 |  | -0.07 |  | -0.02 |  | * |
|  |  | tmin.dry.mean | | -4.63 |  | -10.30 |  | 1.00 |  |  |
|  |  | pr.dry.mean |  | 0.01 |  | -0.10 |  | 0.12 |  |  |
|  |  | tmax.dry.mean | | 0.84 |  | -3.47 |  | 5.14 |  |  |
| Chrysophyllum boukokoense | | | | |  |  |  |  |  |  |
|  |  | intercept |  | 0.02 |  | -0.77 |  | 0.81 |  |  |
|  |  | hunting1 |  | 6.78 |  | -2.94 |  | 16.80 |  |  |
|  |  | logging1 |  | 2.51 |  | -7.16 |  | 12.60 |  |  |
|  |  | Dist |  | 0.28 |  | -0.13 |  | 0.71 |  |  |
|  |  | K |  | -0.04 |  | -0.19 |  | 0.11 |  |  |
|  |  | P |  | 0.12 |  | -1.37 |  | 1.64 |  |  |
|  |  | Ph |  | -4.11 |  | -11.90 |  | 3.41 |  |  |
|  |  | TKN |  | -0.01 |  | -0.03 |  | 0.00 |  |  |
|  |  | tmin.dry.mean | | -0.99 |  | -3.86 |  | 1.85 |  |  |
|  |  | pr.dry.mean |  | 0.00 |  | -0.06 |  | 0.06 |  |  |
|  |  | tmax.dry.mean | | 1.21 |  | -1.05 |  | 3.51 |  |  |
| Chrysophyllum lacourtiana | | | | |  |  |  |  |  |  |
|  |  | intercept |  | 0.75 |  | -0.12 |  | 1.66 |  |  |
|  |  | hunting1 |  | 3.79 |  | -6.47 |  | 14.00 |  |  |
|  |  | logging1 |  | -8.64 |  | -19.90 |  | 1.85 |  |  |
|  |  | Dist |  | -0.35 |  | -0.78 |  | 0.07 |  |  |
|  |  | K |  | -0.14 |  | -0.30 |  | 0.02 |  |  |
|  |  | P |  | 1.23 |  | -0.37 |  | 2.86 |  |  |
|  |  | Ph |  | 5.97 |  | -1.31 |  | 13.50 |  |  |
|  |  | TKN |  | 0.00 |  | -0.02 |  | 0.01 |  |  |
|  |  | tmin.dry.mean | | -1.83 |  | -6.30 |  | 2.04 |  |  |
|  |  | pr.dry.mean |  | -0.04 |  | -0.12 |  | 0.04 |  |  |
|  |  | tmax.dry.mean | | 1.52 |  | -1.34 |  | 4.85 |  |  |
| Cleistopholis patens | | | |  |  |  |  |  |  |  |
|  |  | intercept |  | -0.35 |  | -1.10 |  | 0.38 |  |  |
|  |  | hunting1 |  | 3.22 |  | -6.35 |  | 12.90 |  |  |
|  |  | logging1 |  | 6.04 |  | -3.06 |  | 15.40 |  |  |
|  |  | Dist |  | 0.25 |  | -0.13 |  | 0.65 |  |  |
|  |  | K |  | -0.02 |  | -0.17 |  | 0.13 |  |  |
|  |  | P |  | 0.71 |  | -0.77 |  | 2.23 |  |  |
|  |  | Ph |  | -5.85 |  | -13.00 |  | 1.13 |  |  |
|  |  | TKN |  | -0.01 |  | -0.03 |  | 0.00 |  |  |
|  |  | tmin.dry.mean | | 1.48 |  | -1.20 |  | 4.22 |  |  |
|  |  | pr.dry.mean |  | -0.04 |  | -0.09 |  | 0.02 |  |  |
|  |  | tmax.dry.mean | | -0.17 |  | -2.27 |  | 1.95 |  |  |
| Cola lateritia | | |  |  |  |  |  |  |  |  |
|  |  | intercept |  | 0.53 |  | -0.19 |  | 1.25 |  |  |
|  |  | hunting1 |  | 5.79 |  | -2.95 |  | 14.70 |  |  |
|  |  | logging1 |  | 2.52 |  | -5.66 |  | 10.70 |  |  |
|  |  | Dist |  | 0.15 |  | -0.20 |  | 0.51 |  |  |
|  |  | K |  | -0.02 |  | -0.15 |  | 0.12 |  |  |
|  |  | P |  | -0.02 |  | -1.33 |  | 1.31 |  |  |
|  |  | Ph |  | 3.48 |  | -2.88 |  | 9.89 |  |  |
|  |  | TKN |  | 0.00 |  | -0.02 |  | 0.01 |  |  |
|  |  | tmin.dry.mean | | -0.27 |  | -2.93 |  | 2.34 |  |  |
|  |  | pr.dry.mean |  | -0.02 |  | -0.08 |  | 0.03 |  |  |
|  |  | tmax.dry.mean | | -0.08 |  | -2.08 |  | 1.95 |  |  |
| Dacryodes edulis | | |  |  |  |  |  |  |  |  |
|  |  | intercept |  | -0.26 |  | -1.04 |  | 0.51 |  |  |
|  |  | hunting1 |  | -4.73 |  | -14.90 |  | 5.12 |  |  |
|  |  | logging1 |  | -2.80 |  | -11.90 |  | 6.07 |  |  |
|  |  | Dist |  | -0.21 |  | -0.63 |  | 0.19 |  |  |
|  |  | K |  | 0.05 |  | -0.10 |  | 0.21 |  |  |
|  |  | P |  | -0.66 |  | -2.14 |  | 0.79 |  |  |
|  |  | Ph |  | -0.63 |  | -7.37 |  | 6.14 |  |  |
|  |  | TKN |  | -0.01 |  | -0.02 |  | 0.01 |  |  |
|  |  | tmin.dry.mean | | 0.42 |  | -2.33 |  | 3.10 |  |  |
|  |  | pr.dry.mean |  | 0.02 |  | -0.04 |  | 0.08 |  |  |
|  |  | tmax.dry.mean | | 0.06 |  | -2.01 |  | 2.17 |  |  |
| Desplatsia chrysochlamys | | | | |  |  |  |  |  |  |
|  |  | intercept |  | 0.20 |  | -0.97 |  | 1.38 |  |  |
|  |  | hunting1 |  | -5.72 |  | -20.40 |  | 7.87 |  |  |
|  |  | logging1 |  | -14.40 |  | -26.90 |  | -3.02 |  | * |
|  |  | Dist |  | -0.73 |  | -1.29 |  | -0.23 |  | * |
|  |  | K |  | 0.02 |  | -0.15 |  | 0.19 |  |  |
|  |  | P |  | 0.01 |  | -1.86 |  | 1.88 |  |  |
|  |  | Ph |  | 3.78 |  | -4.85 |  | 12.90 |  |  |
|  |  | TKN |  | -0.01 |  | -0.02 |  | 0.01 |  |  |
|  |  | tmin.dry.mean | | -2.81 |  | -7.46 |  | 1.67 |  |  |
|  |  | pr.dry.mean |  | -0.03 |  | -0.11 |  | 0.06 |  |  |
|  |  | tmax.dry.mean | | 2.70 |  | -0.61 |  | 6.07 |  |  |
| Desplatsia dewevrei | | | |  |  |  |  |  |  |  |
|  |  | intercept |  | 0.65 |  | -0.40 |  | 1.78 |  |  |
|  |  | hunting1 |  | 0.16 |  | -12.70 |  | 13.00 |  |  |
|  |  | logging1 |  | -10.30 |  | -23.20 |  | 1.27 |  |  |
|  |  | Dist |  | -0.45 |  | -0.94 |  | 0.01 |  |  |
|  |  | K |  | 0.04 |  | -0.11 |  | 0.19 |  |  |
|  |  | P |  | -0.02 |  | -1.77 |  | 1.79 |  |  |
|  |  | Ph |  | 7.09 |  | -0.94 |  | 15.70 |  |  |
|  |  | TKN |  | 0.00 |  | -0.01 |  | 0.01 |  |  |
|  |  | tmin.dry.mean | | -1.75 |  | -5.90 |  | 2.26 |  |  |
|  |  | pr.dry.mean |  | -0.03 |  | -0.10 |  | 0.05 |  |  |
|  |  | tmax.dry.mean | | 0.88 |  | -2.21 |  | 4.01 |  |  |
| Dialium pachyphyllum | | | |  |  |  |  |  |  |  |
|  |  | intercept |  | 0.40 |  | -0.30 |  | 1.10 |  |  |
|  |  | hunting1 |  | 7.70 |  | -0.97 |  | 16.50 |  |  |
|  |  | logging1 |  | -3.11 |  | -11.20 |  | 5.02 |  |  |
|  |  | Dist |  | 0.29 |  | -0.06 |  | 0.63 |  |  |
|  |  | K |  | -0.08 |  | -0.22 |  | 0.06 |  |  |
|  |  | P |  | 0.58 |  | -0.75 |  | 1.91 |  |  |
|  |  | Ph |  | -0.44 |  | -6.79 |  | 5.87 |  |  |
|  |  | TKN |  | -0.01 |  | -0.02 |  | 0.01 |  |  |
|  |  | tmin.dry.mean | | -1.58 |  | -4.10 |  | 0.95 |  |  |
|  |  | pr.dry.mean |  | 0.00 |  | -0.05 |  | 0.05 |  |  |
|  |  | tmax.dry.mean | | 1.42 |  | -0.51 |  | 3.36 |  |  |
| Dichostemma glaucescens | | | | |  |  |  |  |  |  |
|  |  | intercept |  | -4.18 |  | -6.31 |  | -2.25 |  | * |
|  |  | hunting1 |  | 25.50 |  | 9.75 |  | 38.40 |  | * |
|  |  | logging1 |  | 28.80 |  | 11.90 |  | 34.10 |  | * |
|  |  | Dist |  | 1.81 |  | 1.19 |  | 2.21 |  | * |
|  |  | K |  | 1.40 |  | 0.87 |  | 1.92 |  | * |
|  |  | P |  | -5.73 |  | -10.50 |  | -1.46 |  | * |
|  |  | Ph |  | -71.70 |  | -97.50 |  | -47.50 |  | * |
|  |  | TKN |  | -0.06 |  | -0.10 |  | -0.03 |  | * |
|  |  | tmin.dry.mean | | -11.60 |  | -21.30 |  | -2.45 |  | * |
|  |  | pr.dry.mean |  | 0.11 |  | -0.06 |  | 0.30 |  |  |
|  |  | tmax.dry.mean | | 11.40 |  | 4.30 |  | 19.00 |  | * |
| Diospyros bipindensis | | | |  |  |  |  |  |  |  |
|  |  | intercept |  | 0.04 |  | -0.68 |  | 0.76 |  |  |
|  |  | hunting1 |  | -0.07 |  | -9.08 |  | 8.98 |  |  |
|  |  | logging1 |  | -2.67 |  | -10.90 |  | 5.54 |  |  |
|  |  | Dist |  | 0.38 |  | 0.02 |  | 0.74 |  | * |
|  |  | K |  | 0.22 |  | 0.08 |  | 0.36 |  | * |
|  |  | P |  | 0.20 |  | -1.14 |  | 1.54 |  |  |
|  |  | Ph |  | -0.12 |  | -6.68 |  | 6.42 |  |  |
|  |  | TKN |  | -0.02 |  | -0.03 |  | 0.00 |  | * |
|  |  | tmin.dry.mean | | -1.51 |  | -4.12 |  | 1.09 |  |  |
|  |  | pr.dry.mean |  | -0.02 |  | -0.07 |  | 0.03 |  |  |
|  |  | tmax.dry.mean | | 0.70 |  | -1.31 |  | 2.68 |  |  |
| Diospyros canaliculata | | | |  |  |  |  |  |  |  |
|  |  | intercept |  | 0.70 |  | -0.24 |  | 1.65 |  |  |
|  |  | hunting1 |  | -5.27 |  | -16.50 |  | 5.96 |  |  |
|  |  | logging1 |  | 2.02 |  | -7.47 |  | 12.10 |  |  |
|  |  | Dist |  | -0.04 |  | -0.46 |  | 0.40 |  |  |
|  |  | K |  | -0.14 |  | -0.35 |  | 0.06 |  |  |
|  |  | P |  | 1.02 |  | -0.93 |  | 2.99 |  |  |
|  |  | Ph |  | 12.60 |  | 3.43 |  | 22.00 |  | * |
|  |  | TKN |  | 0.01 |  | -0.01 |  | 0.02 |  |  |
|  |  | tmin.dry.mean | | 0.68 |  | -3.20 |  | 4.53 |  |  |
|  |  | pr.dry.mean |  | -0.05 |  | -0.13 |  | 0.02 |  |  |
|  |  | tmax.dry.mean | | -1.38 |  | -4.33 |  | 1.55 |  |  |
| Diospyros crassiflora | | | |  |  |  |  |  |  |  |
|  |  | intercept |  | 0.08 |  | -0.77 |  | 0.92 |  |  |
|  |  | hunting1 |  | 5.57 |  | -4.65 |  | 15.90 |  |  |
|  |  | logging1 |  | -10.60 |  | -22.50 |  | 0.37 |  |  |
|  |  | Dist |  | 0.00 |  | -0.40 |  | 0.40 |  |  |
|  |  | K |  | 0.05 |  | -0.11 |  | 0.22 |  |  |
|  |  | P |  | 1.04 |  | -0.55 |  | 2.68 |  |  |
|  |  | Ph |  | -3.91 |  | -11.90 |  | 3.75 |  |  |
|  |  | TKN |  | -0.02 |  | -0.03 |  | 0.00 |  | * |
|  |  | tmin.dry.mean | | -2.17 |  | -5.40 |  | 0.95 |  |  |
|  |  | pr.dry.mean |  | 0.03 |  | -0.03 |  | 0.10 |  |  |
|  |  | tmax.dry.mean | | 1.87 |  | -0.44 |  | 4.30 |  |  |
| Diospyros iturensis | | |  |  |  |  |  |  |  |  |
|  |  | intercept |  | 0.41 |  | -0.31 |  | 1.15 |  |  |
|  |  | hunting1 |  | 5.53 |  | -3.60 |  | 14.80 |  |  |
|  |  | logging1 |  | 2.15 |  | -6.00 |  | 10.30 |  |  |
|  |  | Dist |  | 0.39 |  | 0.03 |  | 0.76 |  | * |
|  |  | K |  | -0.09 |  | -0.23 |  | 0.05 |  |  |
|  |  | P |  | 1.11 |  | -0.25 |  | 2.47 |  |  |
|  |  | Ph |  | 2.22 |  | -4.30 |  | 8.80 |  |  |
|  |  | TKN |  | 0.00 |  | -0.02 |  | 0.01 |  |  |
|  |  | tmin.dry.mean | | 0.28 |  | -2.29 |  | 2.84 |  |  |
|  |  | pr.dry.mean |  | -0.05 |  | -0.10 |  | 0.00 |  |  |
|  |  | tmax.dry.mean | | -0.35 |  | -2.32 |  | 1.61 |  |  |
| Diospyros mannii | | |  |  |  |  |  |  |  |  |
|  |  | intercept |  | -0.04 |  | -0.83 |  | 0.74 |  |  |
|  |  | hunting1 |  | -3.59 |  | -14.40 |  | 6.88 |  |  |
|  |  | logging1 |  | 1.44 |  | -7.12 |  | 10.10 |  |  |
|  |  | Dist |  | 0.05 |  | -0.37 |  | 0.47 |  |  |
|  |  | K |  | -0.01 |  | -0.15 |  | 0.14 |  |  |
|  |  | P |  | -0.72 |  | -2.29 |  | 0.81 |  |  |
|  |  | Ph |  | 1.98 |  | -4.70 |  | 8.79 |  |  |
|  |  | TKN |  | -0.01 |  | -0.02 |  | 0.01 |  |  |
|  |  | tmin.dry.mean | | 0.65 |  | -2.10 |  | 3.39 |  |  |
|  |  | pr.dry.mean |  | 0.03 |  | -0.03 |  | 0.09 |  |  |
|  |  | tmax.dry.mean | | -0.61 |  | -2.69 |  | 1.48 |  |  |
| Discoglypremna caloneura | | | | |  |  |  |  |  |  |
|  |  | intercept |  | -0.32 |  | -1.06 |  | 0.42 |  |  |
|  |  | hunting1 |  | -2.90 |  | -12.10 |  | 6.39 |  |  |
|  |  | logging1 |  | 2.41 |  | -5.81 |  | 10.70 |  |  |
|  |  | Dist |  | -0.10 |  | -0.47 |  | 0.27 |  |  |
|  |  | K |  | -0.02 |  | -0.17 |  | 0.12 |  |  |
|  |  | P |  | -0.26 |  | -1.58 |  | 1.09 |  |  |
|  |  | Ph |  | -2.38 |  | -8.86 |  | 4.08 |  |  |
|  |  | TKN |  | 0.00 |  | -0.01 |  | 0.01 |  |  |
|  |  | tmin.dry.mean | | 0.20 |  | -2.56 |  | 2.95 |  |  |
|  |  | pr.dry.mean |  | -0.03 |  | -0.08 |  | 0.03 |  |  |
|  |  | tmax.dry.mean | | 0.66 |  | -1.39 |  | 2.73 |  |  |
| Drypetes gossweileri | | | |  |  |  |  |  |  |  |
|  |  | intercept |  | 0.32 |  | -0.43 |  | 1.07 |  |  |
|  |  | hunting1 |  | 4.39 |  | -5.57 |  | 14.30 |  |  |
|  |  | logging1 |  | 0.87 |  | -8.34 |  | 10.10 |  |  |
|  |  | Dist |  | 0.32 |  | -0.09 |  | 0.73 |  |  |
|  |  | K |  | -0.05 |  | -0.20 |  | 0.10 |  |  |
|  |  | P |  | 0.40 |  | -1.13 |  | 1.96 |  |  |
|  |  | Ph |  | 1.78 |  | -5.10 |  | 8.68 |  |  |
|  |  | TKN |  | -0.01 |  | -0.03 |  | 0.01 |  |  |
|  |  | tmin.dry.mean | | 0.23 |  | -2.67 |  | 3.17 |  |  |
|  |  | pr.dry.mean |  | 0.03 |  | -0.04 |  | 0.09 |  |  |
|  |  | tmax.dry.mean | | -0.61 |  | -2.87 |  | 1.59 |  |  |
| Drypetes ituriensis | | |  |  |  |  |  |  |  |  |
|  |  | intercept |  | -0.02 |  | -0.98 |  | 0.92 |  |  |
|  |  | hunting1 |  | -1.22 |  | -13.60 |  | 10.40 |  |  |
|  |  | logging1 |  | 9.70 |  | -0.21 |  | 20.20 |  |  |
|  |  | Dist |  | 0.53 |  | 0.07 |  | 1.01 |  | * |
|  |  | K |  | 0.08 |  | -0.09 |  | 0.25 |  |  |
|  |  | P |  | -1.44 |  | -3.36 |  | 0.31 |  |  |
|  |  | Ph |  | 1.29 |  | -6.63 |  | 9.39 |  |  |
|  |  | TKN |  | -0.01 |  | -0.03 |  | 0.01 |  |  |
|  |  | tmin.dry.mean | | -0.13 |  | -3.11 |  | 2.79 |  |  |
|  |  | pr.dry.mean |  | 0.00 |  | -0.07 |  | 0.06 |  |  |
|  |  | tmax.dry.mean | | -0.47 |  | -2.71 |  | 1.79 |  |  |
| Drypetes occidentalis | | | |  |  |  |  |  |  |  |
|  |  | intercept |  | 0.57 |  | -0.20 |  | 1.37 |  |  |
|  |  | hunting1 |  | 7.07 |  | -1.88 |  | 16.20 |  |  |
|  |  | logging1 |  | 3.06 |  | -5.60 |  | 11.70 |  |  |
|  |  | Dist |  | 0.36 |  | -0.02 |  | 0.75 |  |  |
|  |  | K |  | 0.04 |  | -0.10 |  | 0.19 |  |  |
|  |  | P |  | -0.44 |  | -1.83 |  | 0.96 |  |  |
|  |  | Ph |  | 2.62 |  | -4.41 |  | 9.69 |  |  |
|  |  | TKN |  | 0.00 |  | -0.02 |  | 0.01 |  |  |
|  |  | tmin.dry.mean | | -2.48 |  | -5.69 |  | 0.45 |  |  |
|  |  | pr.dry.mean |  | -0.04 |  | -0.10 |  | 0.02 |  |  |
|  |  | tmax.dry.mean | | 1.18 |  | -1.08 |  | 3.65 |  |  |
| Drypetes polyantha | | |  |  |  |  |  |  |  |  |
|  |  | intercept |  | 0.42 |  | -0.33 |  | 1.18 |  |  |
|  |  | hunting1 |  | -0.55 |  | -10.00 |  | 8.87 |  |  |
|  |  | logging1 |  | -0.43 |  | -8.78 |  | 7.97 |  |  |
|  |  | Dist |  | 0.10 |  | -0.27 |  | 0.47 |  |  |
|  |  | K |  | -0.06 |  | -0.22 |  | 0.09 |  |  |
|  |  | P |  | -0.30 |  | -1.75 |  | 1.15 |  |  |
|  |  | Ph |  | 5.14 |  | -1.78 |  | 12.30 |  |  |
|  |  | TKN |  | 0.00 |  | -0.01 |  | 0.01 |  |  |
|  |  | tmin.dry.mean | | -1.72 |  | -4.62 |  | 1.12 |  |  |
|  |  | pr.dry.mean |  | 0.00 |  | -0.06 |  | 0.06 |  |  |
|  |  | tmax.dry.mean | | 0.76 |  | -1.36 |  | 2.93 |  |  |
| Drypetes sp. | | |  |  |  |  |  |  |  |  |
|  |  | intercept |  | 0.15 |  | -0.64 |  | 0.94 |  |  |
|  |  | hunting1 |  | 11.60 |  | 1.13 |  | 22.40 |  | * |
|  |  | logging1 |  | 12.00 |  | 0.19 |  | 25.30 |  | * |
|  |  | Dist |  | 0.67 |  | 0.20 |  | 1.19 |  | * |
|  |  | K |  | -0.17 |  | -0.37 |  | 0.01 |  |  |
|  |  | P |  | 0.80 |  | -0.92 |  | 2.56 |  |  |
|  |  | Ph |  | -4.01 |  | -12.30 |  | 3.91 |  |  |
|  |  | TKN |  | 0.00 |  | -0.02 |  | 0.02 |  |  |
|  |  | tmin.dry.mean | | 0.63 |  | -2.54 |  | 3.80 |  |  |
|  |  | pr.dry.mean |  | 0.01 |  | -0.05 |  | 0.07 |  |  |
|  |  | tmax.dry.mean | | -0.54 |  | -2.84 |  | 1.75 |  |  |
| Duboscia macrocarpa | | | |  |  |  |  |  |  |  |
|  |  | intercept |  | 0.25 |  | -0.53 |  | 1.05 |  |  |
|  |  | hunting1 |  | 3.31 |  | -6.76 |  | 13.70 |  |  |
|  |  | logging1 |  | 0.41 |  | -8.72 |  | 9.68 |  |  |
|  |  | Dist |  | 0.07 |  | -0.31 |  | 0.47 |  |  |
|  |  | K |  | -0.11 |  | -0.26 |  | 0.04 |  |  |
|  |  | P |  | 0.87 |  | -0.69 |  | 2.49 |  |  |
|  |  | Ph |  | 1.48 |  | -5.21 |  | 8.34 |  |  |
|  |  | TKN |  | 0.01 |  | 0.00 |  | 0.02 |  |  |
|  |  | tmin.dry.mean | | 0.48 |  | -2.27 |  | 3.29 |  |  |
|  |  | pr.dry.mean |  | 0.00 |  | -0.06 |  | 0.06 |  |  |
|  |  | tmax.dry.mean | | -0.65 |  | -2.81 |  | 1.46 |  |  |
| Entandrophragma angolense | | | | |  |  |  |  |  |  |
|  |  | intercept |  | -0.34 |  | -1.16 |  | 0.47 |  |  |
|  |  | hunting1 |  | 1.45 |  | -9.10 |  | 11.80 |  |  |
|  |  | logging1 |  | 4.83 |  | -5.07 |  | 15.20 |  |  |
|  |  | Dist |  | 0.08 |  | -0.36 |  | 0.52 |  |  |
|  |  | K |  | 0.12 |  | -0.04 |  | 0.27 |  |  |
|  |  | P |  | -0.02 |  | -1.49 |  | 1.44 |  |  |
|  |  | Ph |  | -4.54 |  | -12.30 |  | 2.92 |  |  |
|  |  | TKN |  | 0.00 |  | -0.01 |  | 0.01 |  |  |
|  |  | tmin.dry.mean | | 0.95 |  | -1.92 |  | 3.85 |  |  |
|  |  | pr.dry.mean |  | -0.02 |  | -0.09 |  | 0.04 |  |  |
|  |  | tmax.dry.mean | | -0.49 |  | -2.66 |  | 1.67 |  |  |
| Entandrophragma candollei | | | | |  |  |  |  |  |  |
|  |  | intercept |  | 0.17 |  | -0.60 |  | 0.96 |  |  |
|  |  | hunting1 |  | 2.49 |  | -7.30 |  | 12.20 |  |  |
|  |  | logging1 |  | 3.45 |  | -5.03 |  | 12.10 |  |  |
|  |  | Dist |  | 0.10 |  | -0.28 |  | 0.48 |  |  |
|  |  | K |  | 0.03 |  | -0.11 |  | 0.17 |  |  |
|  |  | P |  | -0.11 |  | -1.50 |  | 1.29 |  |  |
|  |  | Ph |  | 1.45 |  | -5.35 |  | 8.53 |  |  |
|  |  | TKN |  | 0.00 |  | -0.02 |  | 0.01 |  |  |
|  |  | tmin.dry.mean | | 1.43 |  | -1.19 |  | 4.07 |  |  |
|  |  | pr.dry.mean |  | -0.02 |  | -0.08 |  | 0.03 |  |  |
|  |  | tmax.dry.mean | | -1.13 |  | -3.17 |  | 0.88 |  |  |
| Entandrophragma cylindricum | | | | |  |  |  |  |  |  |
|  |  | intercept |  | 0.27 |  | -0.47 |  | 1.01 |  |  |
|  |  | hunting1 |  | 0.26 |  | -8.70 |  | 9.12 |  |  |
|  |  | logging1 |  | -0.79 |  | -9.08 |  | 7.51 |  |  |
|  |  | Dist |  | 0.13 |  | -0.24 |  | 0.48 |  |  |
|  |  | K |  | -0.04 |  | -0.19 |  | 0.10 |  |  |
|  |  | P |  | 0.00 |  | -1.36 |  | 1.39 |  |  |
|  |  | Ph |  | 3.09 |  | -3.65 |  | 9.93 |  |  |
|  |  | TKN |  | 0.00 |  | -0.02 |  | 0.01 |  |  |
|  |  | tmin.dry.mean | | -0.56 |  | -3.13 |  | 2.02 |  |  |
|  |  | pr.dry.mean |  | 0.01 |  | -0.04 |  | 0.06 |  |  |
|  |  | tmax.dry.mean | | 0.18 |  | -1.81 |  | 2.18 |  |  |
| Entandrophragma utile | | | |  |  |  |  |  |  |  |
|  |  | intercept |  | 0.29 |  | -0.52 |  | 1.09 |  |  |
|  |  | hunting1 |  | -0.45 |  | -10.70 |  | 9.55 |  |  |
|  |  | logging1 |  | -2.90 |  | -12.50 |  | 6.67 |  |  |
|  |  | Dist |  | -0.06 |  | -0.49 |  | 0.36 |  |  |
|  |  | K |  | -0.01 |  | -0.16 |  | 0.14 |  |  |
|  |  | P |  | 0.32 |  | -1.19 |  | 1.81 |  |  |
|  |  | Ph |  | 3.45 |  | -3.63 |  | 10.70 |  |  |
|  |  | TKN |  | -0.01 |  | -0.02 |  | 0.00 |  |  |
|  |  | tmin.dry.mean | | -0.66 |  | -3.70 |  | 2.33 |  |  |
|  |  | pr.dry.mean |  | -0.03 |  | -0.10 |  | 0.03 |  |  |
|  |  | tmax.dry.mean | | 0.45 |  | -1.91 |  | 2.82 |  |  |
| Erythrophleum suaveolens | | | | |  |  |  |  |  |  |
|  |  | intercept |  | 0.84 |  | 0.03 |  | 1.67 |  | * |
|  |  | hunting1 |  | 9.05 |  | -0.23 |  | 18.60 |  |  |
|  |  | logging1 |  | -1.66 |  | -10.40 |  | 6.96 |  |  |
|  |  | Dist |  | 0.21 |  | -0.16 |  | 0.58 |  |  |
|  |  | K |  | -0.05 |  | -0.20 |  | 0.10 |  |  |
|  |  | P |  | -0.30 |  | -1.66 |  | 1.05 |  |  |
|  |  | Ph |  | 5.40 |  | -1.73 |  | 12.80 |  |  |
|  |  | TKN |  | 0.00 |  | -0.01 |  | 0.01 |  |  |
|  |  | tmin.dry.mean | | -0.28 |  | -3.00 |  | 2.35 |  |  |
|  |  | pr.dry.mean |  | 0.00 |  | -0.06 |  | 0.05 |  |  |
|  |  | tmax.dry.mean | | -0.43 |  | -2.48 |  | 1.63 |  |  |
| Fernandoa adolfi friderici | | | | |  |  |  |  |  |  |
|  |  | intercept |  | 0.63 |  | -0.28 |  | 1.55 |  |  |
|  |  | hunting1 |  | 5.61 |  | -6.25 |  | 17.40 |  |  |
|  |  | logging1 |  | 0.99 |  | -9.07 |  | 11.30 |  |  |
|  |  | Dist |  | 0.15 |  | -0.29 |  | 0.60 |  |  |
|  |  | K |  | -0.11 |  | -0.27 |  | 0.05 |  |  |
|  |  | P |  | 0.15 |  | -1.58 |  | 1.94 |  |  |
|  |  | Ph |  | 4.76 |  | -3.12 |  | 12.80 |  |  |
|  |  | TKN |  | 0.00 |  | -0.01 |  | 0.02 |  |  |
|  |  | tmin.dry.mean | | -0.62 |  | -3.63 |  | 2.37 |  |  |
|  |  | pr.dry.mean |  | 0.01 |  | -0.06 |  | 0.07 |  |  |
|  |  | tmax.dry.mean | | -0.13 |  | -2.51 |  | 2.19 |  |  |
| Funtumia elastica | | |  |  |  |  |  |  |  |  |
|  |  | intercept |  | -0.01 |  | -0.72 |  | 0.70 |  |  |
|  |  | hunting1 |  | -5.40 |  | -14.20 |  | 3.23 |  |  |
|  |  | logging1 |  | -5.11 |  | -13.60 |  | 3.16 |  |  |
|  |  | Dist |  | -0.26 |  | -0.63 |  | 0.08 |  |  |
|  |  | K |  | -0.03 |  | -0.17 |  | 0.10 |  |  |
|  |  | P |  | -0.04 |  | -1.35 |  | 1.27 |  |  |
|  |  | Ph |  | 2.58 |  | -3.87 |  | 9.06 |  |  |
|  |  | TKN |  | 0.00 |  | -0.01 |  | 0.01 |  |  |
|  |  | tmin.dry.mean | | 0.16 |  | -2.40 |  | 2.71 |  |  |
|  |  | pr.dry.mean |  | -0.02 |  | -0.07 |  | 0.03 |  |  |
|  |  | tmax.dry.mean | | 0.39 |  | -1.57 |  | 2.35 |  |  |
| Garcinia punctata | | |  |  |  |  |  |  |  |  |
|  |  | intercept |  | 0.15 |  | -0.80 |  | 1.03 |  |  |
|  |  | hunting1 |  | 17.90 |  | 6.78 |  | 27.70 |  | * |
|  |  | logging1 |  | 14.30 |  | -0.01 |  | 28.50 |  |  |
|  |  | Dist |  | 1.19 |  | 0.57 |  | 1.78 |  | * |
|  |  | K |  | 0.27 |  | 0.05 |  | 0.51 |  | * |
|  |  | P |  | -2.19 |  | -4.32 |  | -0.16 |  | * |
|  |  | Ph |  | -7.82 |  | -19.50 |  | 2.48 |  |  |
|  |  | TKN |  | -0.02 |  | -0.05 |  | 0.00 |  | * |
|  |  | tmin.dry.mean | | -1.47 |  | -5.42 |  | 2.20 |  |  |
|  |  | pr.dry.mean |  | 0.06 |  | -0.01 |  | 0.15 |  |  |
|  |  | tmax.dry.mean | | -0.18 |  | -3.20 |  | 2.94 |  |  |
| Greenwayodendron suaveolens | | | | |  |  |  |  |  |  |
|  |  | intercept |  | 1.81 |  | 0.95 |  | 2.63 |  | * |
|  |  | hunting1 |  | 21.50 |  | 11.00 |  | 28.20 |  | * |
|  |  | logging1 |  | -0.51 |  | -11.50 |  | 11.10 |  |  |
|  |  | Dist |  | 0.86 |  | 0.41 |  | 1.31 |  | * |
|  |  | K |  | -0.26 |  | -0.46 |  | -0.08 |  | * |
|  |  | P |  | 2.78 |  | 0.99 |  | 4.46 |  | * |
|  |  | Ph |  | 10.60 |  | 2.14 |  | 19.40 |  | * |
|  |  | TKN |  | -0.01 |  | -0.03 |  | 0.00 |  |  |
|  |  | tmin.dry.mean | | -0.53 |  | -4.01 |  | 2.82 |  |  |
|  |  | pr.dry.mean |  | 0.07 |  | 0.00 |  | 0.14 |  |  |
|  |  | tmax.dry.mean | | -1.42 |  | -3.93 |  | 1.16 |  |  |
| Grossera macrantha | | | |  |  |  |  |  |  |  |
|  |  | intercept |  | -0.01 |  | -2.09 |  | 2.17 |  |  |
|  |  | hunting1 |  | -9.17 |  | -23.90 |  | 10.30 |  |  |
|  |  | logging1 |  | -27.90 |  | -35.60 |  | -9.29 |  | * |
|  |  | Dist |  | 0.09 |  | -0.59 |  | 1.02 |  |  |
|  |  | K |  | 0.47 |  | -0.09 |  | 1.09 |  |  |
|  |  | P |  | -0.81 |  | -5.49 |  | 3.96 |  |  |
|  |  | Ph |  | 1.76 |  | -24.30 |  | 26.80 |  |  |
|  |  | TKN |  | -0.05 |  | -0.09 |  | -0.01 |  | * |
|  |  | tmin.dry.mean | | -4.70 |  | -14.60 |  | 5.07 |  |  |
|  |  | pr.dry.mean |  | -0.07 |  | -0.28 |  | 0.13 |  |  |
|  |  | tmax.dry.mean | | 3.53 |  | -4.03 |  | 11.00 |  |  |
| Guarea cedrata | | |  |  |  |  |  |  |  |  |
|  |  | intercept |  | 0.41 |  | -0.35 |  | 1.17 |  |  |
|  |  | hunting1 |  | 5.67 |  | -4.16 |  | 15.60 |  |  |
|  |  | logging1 |  | -3.81 |  | -12.70 |  | 4.86 |  |  |
|  |  | Dist |  | 0.11 |  | -0.27 |  | 0.49 |  |  |
|  |  | K |  | -0.09 |  | -0.27 |  | 0.07 |  |  |
|  |  | P |  | 0.31 |  | -1.19 |  | 1.86 |  |  |
|  |  | Ph |  | 1.13 |  | -5.75 |  | 8.03 |  |  |
|  |  | TKN |  | 0.00 |  | -0.02 |  | 0.01 |  |  |
|  |  | tmin.dry.mean | | -1.20 |  | -3.96 |  | 1.56 |  |  |
|  |  | pr.dry.mean |  | 0.01 |  | -0.05 |  | 0.06 |  |  |
|  |  | tmax.dry.mean | | 0.91 |  | -1.22 |  | 3.03 |  |  |
| Guarea thompsonii | | |  |  |  |  |  |  |  |  |
|  |  | intercept |  | -0.49 |  | -1.22 |  | 0.24 |  |  |
|  |  | hunting1 |  | 9.19 |  | -0.17 |  | 18.60 |  |  |
|  |  | logging1 |  | 10.30 |  | 1.20 |  | 19.70 |  | * |
|  |  | Dist |  | 0.58 |  | 0.21 |  | 0.98 |  | * |
|  |  | K |  | 0.10 |  | -0.04 |  | 0.24 |  |  |
|  |  | P |  | 0.02 |  | -1.39 |  | 1.46 |  |  |
|  |  | Ph |  | -12.00 |  | -19.10 |  | -5.08 |  | * |
|  |  | TKN |  | -0.01 |  | -0.03 |  | 0.00 |  |  |
|  |  | tmin.dry.mean | | -1.86 |  | -4.54 |  | 0.81 |  |  |
|  |  | pr.dry.mean |  | 0.00 |  | -0.05 |  | 0.06 |  |  |
|  |  | tmax.dry.mean | | 2.06 |  | 0.01 |  | 4.09 |  | * |
| Hannoa klaineana | | |  |  |  |  |  |  |  |  |
|  |  | intercept |  | -0.25 |  | -1.07 |  | 0.56 |  |  |
|  |  | hunting1 |  | -2.12 |  | -11.60 |  | 7.24 |  |  |
|  |  | logging1 |  | -1.37 |  | -11.00 |  | 8.20 |  |  |
|  |  | Dist |  | -0.02 |  | -0.43 |  | 0.39 |  |  |
|  |  | K |  | 0.00 |  | -0.18 |  | 0.16 |  |  |
|  |  | P |  | -0.25 |  | -1.68 |  | 1.17 |  |  |
|  |  | Ph |  | -2.88 |  | -10.80 |  | 4.84 |  |  |
|  |  | TKN |  | 0.00 |  | -0.01 |  | 0.01 |  |  |
|  |  | tmin.dry.mean | | -2.08 |  | -5.01 |  | 0.80 |  |  |
|  |  | pr.dry.mean |  | 0.03 |  | -0.03 |  | 0.09 |  |  |
|  |  | tmax.dry.mean | | 1.72 |  | -0.45 |  | 3.95 |  |  |
| Hexalobus crispiflorus | | | |  |  |  |  |  |  |  |
|  |  | intercept |  | -0.34 |  | -1.14 |  | 0.44 |  |  |
|  |  | hunting1 |  | -4.97 |  | -15.00 |  | 4.67 |  |  |
|  |  | logging1 |  | -0.59 |  | -8.83 |  | 7.67 |  |  |
|  |  | Dist |  | -0.16 |  | -0.55 |  | 0.22 |  |  |
|  |  | K |  | -0.02 |  | -0.16 |  | 0.13 |  |  |
|  |  | P |  | -0.19 |  | -1.55 |  | 1.16 |  |  |
|  |  | Ph |  | -1.39 |  | -8.33 |  | 5.40 |  |  |
|  |  | TKN |  | 0.00 |  | -0.01 |  | 0.01 |  |  |
|  |  | tmin.dry.mean | | 0.53 |  | -2.06 |  | 3.10 |  |  |
|  |  | pr.dry.mean |  | 0.03 |  | -0.03 |  | 0.08 |  |  |
|  |  | tmax.dry.mean | | 0.06 |  | -1.96 |  | 2.07 |  |  |
| Irvingia grandifolia | | |  |  |  |  |  |  |  |  |
|  |  | intercept |  | 0.57 |  | -0.38 |  | 1.57 |  |  |
|  |  | hunting1 |  | 4.97 |  | -5.83 |  | 16.00 |  |  |
|  |  | logging1 |  | -6.07 |  | -16.00 |  | 3.62 |  |  |
|  |  | Dist |  | -0.01 |  | -0.43 |  | 0.40 |  |  |
|  |  | K |  | 0.01 |  | -0.18 |  | 0.19 |  |  |
|  |  | P |  | 0.90 |  | -0.79 |  | 2.64 |  |  |
|  |  | Ph |  | 4.50 |  | -4.39 |  | 13.50 |  |  |
|  |  | TKN |  | 0.00 |  | -0.01 |  | 0.01 |  |  |
|  |  | tmin.dry.mean | | 0.94 |  | -2.03 |  | 4.00 |  |  |
|  |  | pr.dry.mean |  | -0.03 |  | -0.09 |  | 0.03 |  |  |
|  |  | tmax.dry.mean | | -1.31 |  | -3.83 |  | 1.10 |  |  |
| Isolona hexaloba | | |  |  |  |  |  |  |  |  |
|  |  | intercept |  | 0.57 |  | -0.26 |  | 1.44 |  |  |
|  |  | hunting1 |  | 11.70 |  | 1.97 |  | 21.80 |  | * |
|  |  | logging1 |  | 5.06 |  | -4.14 |  | 14.50 |  |  |
|  |  | Dist |  | 0.40 |  | 0.02 |  | 0.79 |  | * |
|  |  | K |  | -0.02 |  | -0.17 |  | 0.12 |  |  |
|  |  | P |  | 0.04 |  | -1.39 |  | 1.50 |  |  |
|  |  | Ph |  | 1.50 |  | -6.32 |  | 9.46 |  |  |
|  |  | TKN |  | 0.00 |  | -0.02 |  | 0.01 |  |  |
|  |  | tmin.dry.mean | | 1.70 |  | -1.20 |  | 4.66 |  |  |
|  |  | pr.dry.mean |  | -0.03 |  | -0.09 |  | 0.03 |  |  |
|  |  | tmax.dry.mean | | -1.62 |  | -4.09 |  | 0.72 |  |  |
| Keayodendron bridelioides | | | | |  |  |  |  |  |  |
|  |  | intercept |  | 0.32 |  | -0.56 |  | 1.21 |  |  |
|  |  | hunting1 |  | 5.33 |  | -7.71 |  | 17.80 |  |  |
|  |  | logging1 |  | 9.95 |  | -2.16 |  | 24.00 |  |  |
|  |  | Dist |  | 0.13 |  | -0.43 |  | 0.68 |  |  |
|  |  | K |  | -0.10 |  | -0.25 |  | 0.05 |  |  |
|  |  | P |  | 0.50 |  | -1.13 |  | 2.15 |  |  |
|  |  | Ph |  | 1.35 |  | -6.39 |  | 9.00 |  |  |
|  |  | TKN |  | 0.00 |  | -0.01 |  | 0.02 |  |  |
|  |  | tmin.dry.mean | | -0.67 |  | -4.97 |  | 3.52 |  |  |
|  |  | pr.dry.mean |  | -0.01 |  | -0.09 |  | 0.07 |  |  |
|  |  | tmax.dry.mean | | 0.06 |  | -2.99 |  | 3.24 |  |  |
| Klainedoxa gabonensis | | | |  |  |  |  |  |  |  |
|  |  | intercept |  | -0.49 |  | -1.34 |  | 0.33 |  |  |
|  |  | hunting1 |  | -2.41 |  | -13.30 |  | 7.97 |  |  |
|  |  | logging1 |  | 1.54 |  | -7.49 |  | 10.70 |  |  |
|  |  | Dist |  | -0.17 |  | -0.60 |  | 0.26 |  |  |
|  |  | K |  | 0.01 |  | -0.16 |  | 0.18 |  |  |
|  |  | P |  | -0.53 |  | -2.10 |  | 1.00 |  |  |
|  |  | Ph |  | -4.84 |  | -12.60 |  | 2.62 |  |  |
|  |  | TKN |  | 0.00 |  | -0.01 |  | 0.01 |  |  |
|  |  | tmin.dry.mean | | 0.47 |  | -2.42 |  | 3.34 |  |  |
|  |  | pr.dry.mean |  | -0.01 |  | -0.07 |  | 0.05 |  |  |
|  |  | tmax.dry.mean | | 0.60 |  | -1.61 |  | 2.84 |  |  |
| Lecaniodiscus cupanioides | | | | |  |  |  |  |  |  |
|  |  | intercept |  | 0.36 |  | -0.38 |  | 1.11 |  |  |
|  |  | hunting1 |  | 4.17 |  | -5.31 |  | 13.80 |  |  |
|  |  | logging1 |  | 0.64 |  | -8.29 |  | 9.71 |  |  |
|  |  | Dist |  | 0.14 |  | -0.24 |  | 0.51 |  |  |
|  |  | K |  | -0.03 |  | -0.17 |  | 0.11 |  |  |
|  |  | P |  | 0.05 |  | -1.41 |  | 1.54 |  |  |
|  |  | Ph |  | 2.30 |  | -4.28 |  | 8.98 |  |  |
|  |  | TKN |  | 0.00 |  | -0.01 |  | 0.01 |  |  |
|  |  | tmin.dry.mean | | -0.08 |  | -2.75 |  | 2.54 |  |  |
|  |  | pr.dry.mean |  | 0.01 |  | -0.04 |  | 0.07 |  |  |
|  |  | tmax.dry.mean | | -0.40 |  | -2.41 |  | 1.64 |  |  |
| Lepidobotrys staudtii | | | |  |  |  |  |  |  |  |
|  |  | intercept |  | 0.13 |  | -0.84 |  | 1.14 |  |  |
|  |  | hunting1 |  | 0.40 |  | -13.30 |  | 13.60 |  |  |
|  |  | logging1 |  | -11.10 |  | -23.40 |  | 0.03 |  |  |
|  |  | Dist |  | -0.41 |  | -0.94 |  | 0.06 |  |  |
|  |  | K |  | 0.05 |  | -0.10 |  | 0.21 |  |  |
|  |  | P |  | 0.38 |  | -1.41 |  | 2.27 |  |  |
|  |  | Ph |  | 0.92 |  | -6.74 |  | 9.00 |  |  |
|  |  | TKN |  | -0.01 |  | -0.02 |  | 0.00 |  |  |
|  |  | tmin.dry.mean | | 0.50 |  | -2.82 |  | 4.06 |  |  |
|  |  | pr.dry.mean |  | 0.07 |  | 0.00 |  | 0.15 |  |  |
|  |  | tmax.dry.mean | | -0.40 |  | -2.99 |  | 2.02 |  |  |
| Lindackeria dentata | | |  |  |  |  |  |  |  |  |
|  |  | intercept |  | 0.68 |  | -0.15 |  | 1.51 |  |  |
|  |  | hunting1 |  | 8.44 |  | -1.57 |  | 18.50 |  |  |
|  |  | logging1 |  | -1.31 |  | -10.80 |  | 8.08 |  |  |
|  |  | Dist |  | 0.10 |  | -0.29 |  | 0.49 |  |  |
|  |  | K |  | -0.05 |  | -0.21 |  | 0.12 |  |  |
|  |  | P |  | 0.66 |  | -0.91 |  | 2.23 |  |  |
|  |  | Ph |  | 4.54 |  | -2.92 |  | 12.10 |  |  |
|  |  | TKN |  | -0.01 |  | -0.02 |  | 0.01 |  |  |
|  |  | tmin.dry.mean | | 2.61 |  | -0.48 |  | 5.77 |  |  |
|  |  | pr.dry.mean |  | -0.06 |  | -0.13 |  | 0.00 |  | * |
|  |  | tmax.dry.mean | | -1.96 |  | -4.48 |  | 0.52 |  |  |
| Lovoa trichilioides | | |  |  |  |  |  |  |  |  |
|  |  | intercept |  | -0.12 |  | -0.87 |  | 0.65 |  |  |
|  |  | hunting1 |  | 1.24 |  | -8.40 |  | 10.80 |  |  |
|  |  | logging1 |  | 1.12 |  | -8.06 |  | 10.30 |  |  |
|  |  | Dist |  | 0.16 |  | -0.22 |  | 0.54 |  |  |
|  |  | K |  | 0.04 |  | -0.11 |  | 0.18 |  |  |
|  |  | P |  | -0.38 |  | -1.88 |  | 1.13 |  |  |
|  |  | Ph |  | -2.75 |  | -9.76 |  | 4.17 |  |  |
|  |  | TKN |  | 0.00 |  | -0.01 |  | 0.01 |  |  |
|  |  | tmin.dry.mean | | -1.72 |  | -4.56 |  | 1.05 |  |  |
|  |  | pr.dry.mean |  | 0.04 |  | -0.02 |  | 0.09 |  |  |
|  |  | tmax.dry.mean | | 1.11 |  | -0.95 |  | 3.21 |  |  |
| Macaranga barteri | | |  |  |  |  |  |  |  |  |
|  |  | intercept |  | -0.31 |  | -1.04 |  | 0.42 |  |  |
|  |  | hunting1 |  | -0.74 |  | -9.64 |  | 8.24 |  |  |
|  |  | logging1 |  | 1.46 |  | -6.97 |  | 9.91 |  |  |
|  |  | Dist |  | 0.09 |  | -0.27 |  | 0.45 |  |  |
|  |  | K |  | -0.01 |  | -0.15 |  | 0.13 |  |  |
|  |  | P |  | -0.33 |  | -1.68 |  | 1.03 |  |  |
|  |  | Ph |  | -3.54 |  | -10.30 |  | 3.13 |  |  |
|  |  | TKN |  | -0.01 |  | -0.02 |  | 0.00 |  |  |
|  |  | tmin.dry.mean | | 0.43 |  | -2.15 |  | 3.01 |  |  |
|  |  | pr.dry.mean |  | 0.00 |  | -0.06 |  | 0.05 |  |  |
|  |  | tmax.dry.mean | | 0.52 |  | -1.47 |  | 2.51 |  |  |
| Macaranga spinosa | | |  |  |  |  |  |  |  |  |
|  |  | intercept |  | -0.19 |  | -1.55 |  | 0.94 |  |  |
|  |  | hunting1 |  | 28.10 |  | 14.20 |  | 41.10 |  | * |
|  |  | logging1 |  | 20.90 |  | 8.57 |  | 31.40 |  | * |
|  |  | Dist |  | 1.48 |  | 0.86 |  | 2.03 |  | * |
|  |  | K |  | -0.04 |  | -0.31 |  | 0.26 |  |  |
|  |  | P |  | 1.59 |  | -0.73 |  | 4.03 |  |  |
|  |  | Ph |  | -18.60 |  | -35.20 |  | -5.58 |  | * |
|  |  | TKN |  | -0.03 |  | -0.06 |  | -0.01 |  | * |
|  |  | tmin.dry.mean | | -1.25 |  | -5.20 |  | 2.64 |  |  |
|  |  | pr.dry.mean |  | -0.03 |  | -0.12 |  | 0.05 |  |  |
|  |  | tmax.dry.mean | | 1.77 |  | -1.35 |  | 5.18 |  |  |
| Massularia acuminata | | | |  |  |  |  |  |  |  |
|  |  | intercept |  | -0.08 |  | -0.97 |  | 0.83 |  |  |
|  |  | hunting1 |  | 0.15 |  | -11.60 |  | 11.60 |  |  |
|  |  | logging1 |  | 3.72 |  | -6.22 |  | 14.10 |  |  |
|  |  | Dist |  | 0.26 |  | -0.18 |  | 0.71 |  |  |
|  |  | K |  | -0.02 |  | -0.19 |  | 0.14 |  |  |
|  |  | P |  | -0.15 |  | -1.83 |  | 1.53 |  |  |
|  |  | Ph |  | -0.53 |  | -8.48 |  | 7.70 |  |  |
|  |  | TKN |  | 0.00 |  | -0.01 |  | 0.02 |  |  |
|  |  | tmin.dry.mean | | 0.31 |  | -2.70 |  | 3.33 |  |  |
|  |  | pr.dry.mean |  | 0.05 |  | -0.02 |  | 0.11 |  |  |
|  |  | tmax.dry.mean | | -0.79 |  | -3.16 |  | 1.55 |  |  |
| Monodora tenuifolia | | | |  |  |  |  |  |  |  |
|  |  | intercept |  | 0.28 |  | -0.54 |  | 1.11 |  |  |
|  |  | hunting1 |  | 7.72 |  | -2.01 |  | 17.70 |  |  |
|  |  | logging1 |  | -0.55 |  | -10.00 |  | 9.02 |  |  |
|  |  | Dist |  | 0.25 |  | -0.14 |  | 0.64 |  |  |
|  |  | K |  | -0.04 |  | -0.18 |  | 0.10 |  |  |
|  |  | P |  | 0.89 |  | -0.57 |  | 2.41 |  |  |
|  |  | Ph |  | -1.37 |  | -8.73 |  | 6.11 |  |  |
|  |  | TKN |  | -0.01 |  | -0.02 |  | 0.01 |  |  |
|  |  | tmin.dry.mean | | -0.90 |  | -3.60 |  | 1.80 |  |  |
|  |  | pr.dry.mean |  | 0.01 |  | -0.04 |  | 0.07 |  |  |
|  |  | tmax.dry.mean | | 0.47 |  | -1.62 |  | 2.57 |  |  |
| Myrianthus arboreus | | | |  |  |  |  |  |  |  |
|  |  | intercept |  | 0.17 |  | -0.91 |  | 1.25 |  |  |
|  |  | hunting1 |  | 1.59 |  | -10.40 |  | 13.00 |  |  |
|  |  | logging1 |  | -1.04 |  | -11.50 |  | 9.76 |  |  |
|  |  | Dist |  | -0.66 |  | -1.13 |  | -0.20 |  | * |
|  |  | K |  | 0.09 |  | -0.06 |  | 0.24 |  |  |
|  |  | P |  | 0.49 |  | -1.16 |  | 2.15 |  |  |
|  |  | Ph |  | -1.41 |  | -9.90 |  | 7.10 |  |  |
|  |  | TKN |  | 0.00 |  | -0.02 |  | 0.01 |  |  |
|  |  | tmin.dry.mean | | -5.62 |  | -11.40 |  | -0.13 |  | * |
|  |  | pr.dry.mean |  | -0.13 |  | -0.25 |  | -0.03 |  | * |
|  |  | tmax.dry.mean | | 5.17 |  | 1.13 |  | 9.39 |  | * |
| Nesogordonia kabingaensis | | | | |  |  |  |  |  |  |
|  |  | intercept |  | -0.43 |  | -1.15 |  | 0.28 |  |  |
|  |  | hunting1 |  | 0.57 |  | -8.20 |  | 9.48 |  |  |
|  |  | logging1 |  | 4.08 |  | -4.10 |  | 12.30 |  |  |
|  |  | Dist |  | 0.13 |  | -0.22 |  | 0.48 |  |  |
|  |  | K |  | 0.20 |  | 0.06 |  | 0.34 |  | * |
|  |  | P |  | 0.32 |  | -1.02 |  | 1.66 |  |  |
|  |  | Ph |  | -6.03 |  | -12.50 |  | 0.48 |  |  |
|  |  | TKN |  | -0.01 |  | -0.02 |  | 0.00 |  | * |
|  |  | tmin.dry.mean | | -0.70 |  | -3.27 |  | 1.89 |  |  |
|  |  | pr.dry.mean |  | -0.03 |  | -0.08 |  | 0.03 |  |  |
|  |  | tmax.dry.mean | | 0.85 |  | -1.13 |  | 2.82 |  |  |
| Ongokea gore | | |  |  |  |  |  |  |  |  |
|  |  | intercept |  | 0.01 |  | -0.84 |  | 0.87 |  |  |
|  |  | hunting1 |  | 2.70 |  | -8.11 |  | 13.40 |  |  |
|  |  | logging1 |  | 2.97 |  | -6.62 |  | 12.50 |  |  |
|  |  | Dist |  | 0.29 |  | -0.14 |  | 0.72 |  |  |
|  |  | K |  | 0.07 |  | -0.09 |  | 0.23 |  |  |
|  |  | P |  | 0.37 |  | -1.25 |  | 2.00 |  |  |
|  |  | Ph |  | -1.02 |  | -8.08 |  | 5.96 |  |  |
|  |  | TKN |  | -0.01 |  | -0.02 |  | 0.00 |  |  |
|  |  | tmin.dry.mean | | 0.74 |  | -2.31 |  | 3.76 |  |  |
|  |  | pr.dry.mean |  | -0.07 |  | -0.14 |  | -0.01 |  | * |
|  |  | tmax.dry.mean | | -0.44 |  | -2.68 |  | 1.83 |  |  |
| Pancovia laurentii | | |  |  |  |  |  |  |  |  |
|  |  | intercept |  | 0.16 |  | -0.60 |  | 0.91 |  |  |
|  |  | hunting1 |  | 9.20 |  | -0.41 |  | 19.20 |  |  |
|  |  | logging1 |  | 2.26 |  | -6.91 |  | 11.30 |  |  |
|  |  | Dist |  | 0.54 |  | 0.13 |  | 0.98 |  | * |
|  |  | K |  | -0.01 |  | -0.16 |  | 0.14 |  |  |
|  |  | P |  | 0.66 |  | -0.80 |  | 2.14 |  |  |
|  |  | Ph |  | -3.30 |  | -10.20 |  | 3.54 |  |  |
|  |  | TKN |  | -0.02 |  | -0.04 |  | 0.00 |  | * |
|  |  | tmin.dry.mean | | -0.01 |  | -2.70 |  | 2.69 |  |  |
|  |  | pr.dry.mean |  | 0.04 |  | -0.02 |  | 0.10 |  |  |
|  |  | tmax.dry.mean | | -0.16 |  | -2.24 |  | 1.90 |  |  |
| Pancovia pedicellaris | | | |  |  |  |  |  |  |  |
|  |  | intercept |  | -0.15 |  | -1.04 |  | 0.70 |  |  |
|  |  | hunting1 |  | 0.68 |  | -10.70 |  | 11.90 |  |  |
|  |  | logging1 |  | -1.02 |  | -10.20 |  | 7.97 |  |  |
|  |  | Dist |  | 0.36 |  | -0.05 |  | 0.78 |  |  |
|  |  | K |  | -0.07 |  | -0.22 |  | 0.09 |  |  |
|  |  | P |  | 1.42 |  | -0.20 |  | 3.12 |  |  |
|  |  | Ph |  | -2.91 |  | -10.30 |  | 4.26 |  |  |
|  |  | TKN |  | 0.00 |  | -0.02 |  | 0.02 |  |  |
|  |  | tmin.dry.mean | | -0.97 |  | -3.78 |  | 1.83 |  |  |
|  |  | pr.dry.mean |  | 0.00 |  | -0.06 |  | 0.06 |  |  |
|  |  | tmax.dry.mean | | 0.74 |  | -1.36 |  | 2.89 |  |  |
| Panda oleosa | | |  |  |  |  |  |  |  |  |
|  |  | intercept |  | 0.33 |  | -0.39 |  | 1.03 |  |  |
|  |  | hunting1 |  | 3.19 |  | -5.71 |  | 11.90 |  |  |
|  |  | logging1 |  | -3.64 |  | -11.80 |  | 4.55 |  |  |
|  |  | Dist |  | -0.11 |  | -0.46 |  | 0.24 |  |  |
|  |  | K |  | -0.12 |  | -0.26 |  | 0.02 |  |  |
|  |  | P |  | 0.95 |  | -0.38 |  | 2.30 |  |  |
|  |  | Ph |  | 2.27 |  | -4.09 |  | 8.64 |  |  |
|  |  | TKN |  | 0.00 |  | -0.01 |  | 0.01 |  |  |
|  |  | tmin.dry.mean | | 1.06 |  | -1.52 |  | 3.66 |  |  |
|  |  | pr.dry.mean |  | -0.01 |  | -0.06 |  | 0.04 |  |  |
|  |  | tmax.dry.mean | | -0.40 |  | -2.36 |  | 1.56 |  |  |
| Pausinystalia macroceras | | | | |  |  |  |  |  |  |
|  |  | intercept |  | 0.67 |  | -0.08 |  | 1.42 |  |  |
|  |  | hunting1 |  | 5.20 |  | -3.89 |  | 14.40 |  |  |
|  |  | logging1 |  | -1.59 |  | -9.93 |  | 6.70 |  |  |
|  |  | Dist |  | 0.29 |  | -0.08 |  | 0.66 |  |  |
|  |  | K |  | -0.07 |  | -0.22 |  | 0.07 |  |  |
|  |  | P |  | -0.03 |  | -1.40 |  | 1.35 |  |  |
|  |  | Ph |  | 5.00 |  | -1.85 |  | 11.90 |  |  |
|  |  | TKN |  | -0.01 |  | -0.03 |  | 0.00 |  | * |
|  |  | tmin.dry.mean | | -1.32 |  | -3.89 |  | 1.25 |  |  |
|  |  | pr.dry.mean |  | 0.03 |  | -0.02 |  | 0.08 |  |  |
|  |  | tmax.dry.mean | | 0.51 |  | -1.48 |  | 2.51 |  |  |
| Petersianthus macrocarpus | | | | |  |  |  |  |  |  |
|  |  | intercept |  | -0.08 |  | -0.78 |  | 0.63 |  |  |
|  |  | hunting1 |  | 9.89 |  | 1.13 |  | 18.80 |  | * |
|  |  | logging1 |  | 1.83 |  | -6.59 |  | 10.20 |  |  |
|  |  | Dist |  | 0.24 |  | -0.10 |  | 0.59 |  |  |
|  |  | K |  | 0.14 |  | 0.00 |  | 0.28 |  |  |
|  |  | P |  | 1.06 |  | -0.26 |  | 2.40 |  |  |
|  |  | Ph |  | -6.30 |  | -12.60 |  | 0.08 |  |  |
|  |  | TKN |  | -0.02 |  | -0.03 |  | -0.01 |  | * |
|  |  | tmin.dry.mean | | 1.65 |  | -0.89 |  | 4.19 |  |  |
|  |  | pr.dry.mean |  | -0.03 |  | -0.08 |  | 0.02 |  |  |
|  |  | tmax.dry.mean | | -0.70 |  | -2.65 |  | 1.24 |  |  |
| Phyllocosmus africanus | | | |  |  |  |  |  |  |  |
|  |  | intercept |  | 0.22 |  | -0.59 |  | 1.03 |  |  |
|  |  | hunting1 |  | -1.32 |  | -11.60 |  | 8.49 |  |  |
|  |  | logging1 |  | -2.42 |  | -11.20 |  | 6.31 |  |  |
|  |  | Dist |  | -0.17 |  | -0.59 |  | 0.24 |  |  |
|  |  | K |  | -0.04 |  | -0.21 |  | 0.12 |  |  |
|  |  | P |  | -0.64 |  | -2.16 |  | 0.85 |  |  |
|  |  | Ph |  | 2.54 |  | -4.52 |  | 9.63 |  |  |
|  |  | TKN |  | 0.00 |  | -0.01 |  | 0.01 |  |  |
|  |  | tmin.dry.mean | | -2.45 |  | -5.71 |  | 0.75 |  |  |
|  |  | pr.dry.mean |  | 0.01 |  | -0.05 |  | 0.07 |  |  |
|  |  | tmax.dry.mean | | 1.85 |  | -0.54 |  | 4.30 |  |  |
| Picralima nitida | | |  |  |  |  |  |  |  |  |
|  |  | intercept |  | -0.06 |  | -0.90 |  | 0.77 |  |  |
|  |  | hunting1 |  | -4.31 |  | -15.80 |  | 6.60 |  |  |
|  |  | logging1 |  | -1.13 |  | -10.30 |  | 7.98 |  |  |
|  |  | Dist |  | -0.31 |  | -0.75 |  | 0.13 |  |  |
|  |  | K |  | -0.09 |  | -0.26 |  | 0.07 |  |  |
|  |  | P |  | 0.11 |  | -1.47 |  | 1.72 |  |  |
|  |  | Ph |  | 1.63 |  | -5.54 |  | 8.76 |  |  |
|  |  | TKN |  | 0.00 |  | -0.01 |  | 0.01 |  |  |
|  |  | tmin.dry.mean | | 0.34 |  | -3.14 |  | 3.80 |  |  |
|  |  | pr.dry.mean |  | 0.00 |  | -0.07 |  | 0.06 |  |  |
|  |  | tmax.dry.mean | | 0.28 |  | -2.21 |  | 2.83 |  |  |
| Pteleopsis hylodendron | | | |  |  |  |  |  |  |  |
|  |  | intercept |  | -0.24 |  | -1.10 |  | 0.60 |  |  |
|  |  | hunting1 |  | 0.33 |  | -9.79 |  | 10.40 |  |  |
|  |  | logging1 |  | -5.10 |  | -14.20 |  | 3.89 |  |  |
|  |  | Dist |  | -0.13 |  | -0.53 |  | 0.26 |  |  |
|  |  | K |  | -0.02 |  | -0.19 |  | 0.15 |  |  |
|  |  | P |  | 0.47 |  | -1.11 |  | 2.12 |  |  |
|  |  | Ph |  | -3.68 |  | -11.70 |  | 3.88 |  |  |
|  |  | TKN |  | 0.00 |  | -0.01 |  | 0.01 |  |  |
|  |  | tmin.dry.mean | | 0.36 |  | -2.39 |  | 3.18 |  |  |
|  |  | pr.dry.mean |  | 0.02 |  | -0.04 |  | 0.07 |  |  |
|  |  | tmax.dry.mean | | 0.40 |  | -1.82 |  | 2.62 |  |  |
| Pterocarpus soyauxii | | | |  |  |  |  |  |  |  |
|  |  | intercept |  | -0.24 |  | -0.95 |  | 0.47 |  |  |
|  |  | hunting1 |  | -0.01 |  | -8.96 |  | 8.82 |  |  |
|  |  | logging1 |  | 0.96 |  | -7.18 |  | 9.09 |  |  |
|  |  | Dist |  | 0.00 |  | -0.36 |  | 0.35 |  |  |
|  |  | K |  | 0.03 |  | -0.12 |  | 0.17 |  |  |
|  |  | P |  | 0.01 |  | -1.33 |  | 1.34 |  |  |
|  |  | Ph |  | -3.11 |  | -9.47 |  | 3.25 |  |  |
|  |  | TKN |  | 0.00 |  | -0.01 |  | 0.01 |  |  |
|  |  | tmin.dry.mean | | -0.03 |  | -2.60 |  | 2.57 |  |  |
|  |  | pr.dry.mean |  | 0.00 |  | -0.05 |  | 0.05 |  |  |
|  |  | tmax.dry.mean | | 0.48 |  | -1.47 |  | 2.46 |  |  |
| Radlkofera calodendron | | | | |  |  |  |  |  |  |
|  |  | intercept |  | -0.19 |  | -0.99 |  | 0.60 |  |  |
|  |  | hunting1 |  | 1.85 |  | -7.91 |  | 11.80 |  |  |
|  |  | logging1 |  | -9.16 |  | -18.20 |  | -0.30 |  | * |
|  |  | Dist |  | -0.12 |  | -0.50 |  | 0.26 |  |  |
|  |  | K |  | -0.11 |  | -0.29 |  | 0.05 |  |  |
|  |  | P |  | 0.77 |  | -0.77 |  | 2.36 |  |  |
|  |  | Ph |  | -4.57 |  | -12.10 |  | 2.70 |  |  |
|  |  | TKN |  | -0.01 |  | -0.02 |  | 0.01 |  |  |
|  |  | tmin.dry.mean | | 0.22 |  | -2.46 |  | 2.90 |  |  |
|  |  | pr.dry.mean |  | 0.00 |  | -0.05 |  | 0.06 |  |  |
|  |  | tmax.dry.mean | | 1.27 |  | -0.92 |  | 3.48 |  |  |
| Rinorea oblongifolia | | | |  |  |  |  |  |  |  |
|  |  | intercept |  | -0.91 |  | -1.91 |  | 0.16 |  |  |
|  |  | hunting1 |  | -27.60 |  | -36.40 |  | -15.40 |  | * |
|  |  | logging1 |  | -14.30 |  | -28.20 |  | -2.40 |  | * |
|  |  | Dist |  | -1.03 |  | -1.57 |  | -0.49 |  | * |
|  |  | K |  | -0.13 |  | -0.43 |  | 0.14 |  |  |
|  |  | P |  | -2.09 |  | -4.22 |  | 0.10 |  |  |
|  |  | Ph |  | 3.47 |  | -7.35 |  | 14.50 |  |  |
|  |  | TKN |  | 0.01 |  | -0.01 |  | 0.03 |  |  |
|  |  | tmin.dry.mean | | 0.32 |  | -4.11 |  | 4.75 |  |  |
|  |  | pr.dry.mean |  | 0.07 |  | -0.02 |  | 0.16 |  |  |
|  |  | tmax.dry.mean | | 1.27 |  | -1.91 |  | 4.59 |  |  |
| Santiria trimera | | |  |  |  |  |  |  |  |  |
|  |  | intercept |  | 0.12 |  | -0.63 |  | 0.87 |  |  |
|  |  | hunting1 |  | 5.58 |  | -3.51 |  | 14.80 |  |  |
|  |  | logging1 |  | 1.49 |  | -7.36 |  | 10.30 |  |  |
|  |  | Dist |  | 0.32 |  | -0.05 |  | 0.70 |  |  |
|  |  | K |  | 0.08 |  | -0.07 |  | 0.23 |  |  |
|  |  | P |  | -0.09 |  | -1.58 |  | 1.40 |  |  |
|  |  | Ph |  | -1.36 |  | -8.12 |  | 5.41 |  |  |
|  |  | TKN |  | 0.00 |  | -0.01 |  | 0.01 |  |  |
|  |  | tmin.dry.mean | | 0.48 |  | -2.22 |  | 3.20 |  |  |
|  |  | pr.dry.mean |  | 0.01 |  | -0.05 |  | 0.06 |  |  |
|  |  | tmax.dry.mean | | -0.83 |  | -2.97 |  | 1.26 |  |  |
| Scottellia klaineana | | |  |  |  |  |  |  |  |  |
|  |  | intercept |  | 0.28 |  | -0.50 |  | 1.06 |  |  |
|  |  | hunting1 |  | 3.24 |  | -5.94 |  | 12.50 |  |  |
|  |  | logging1 |  | -1.70 |  | -10.60 |  | 7.19 |  |  |
|  |  | Dist |  | 0.31 |  | -0.07 |  | 0.71 |  |  |
|  |  | K |  | 0.00 |  | -0.16 |  | 0.16 |  |  |
|  |  | P |  | -0.52 |  | -1.98 |  | 0.96 |  |  |
|  |  | Ph |  | 1.09 |  | -6.40 |  | 8.49 |  |  |
|  |  | TKN |  | -0.01 |  | -0.02 |  | 0.01 |  |  |
|  |  | tmin.dry.mean | | -1.64 |  | -4.30 |  | 1.03 |  |  |
|  |  | pr.dry.mean |  | 0.02 |  | -0.04 |  | 0.08 |  |  |
|  |  | tmax.dry.mean | | 0.81 |  | -1.25 |  | 2.91 |  |  |
| Sterculia oblonga | | |  |  |  |  |  |  |  |  |
|  |  | intercept |  | 0.46 |  | -0.27 |  | 1.20 |  |  |
|  |  | hunting1 |  | 2.85 |  | -6.18 |  | 11.90 |  |  |
|  |  | logging1 |  | -6.87 |  | -15.80 |  | 1.96 |  |  |
|  |  | Dist |  | -0.19 |  | -0.56 |  | 0.17 |  |  |
|  |  | K |  | -0.08 |  | -0.22 |  | 0.06 |  |  |
|  |  | P |  | 1.34 |  | -0.04 |  | 2.72 |  |  |
|  |  | Ph |  | 3.57 |  | -2.97 |  | 10.10 |  |  |
|  |  | TKN |  | -0.01 |  | -0.02 |  | 0.01 |  |  |
|  |  | tmin.dry.mean | | -0.18 |  | -2.99 |  | 2.60 |  |  |
|  |  | pr.dry.mean |  | -0.02 |  | -0.08 |  | 0.04 |  |  |
|  |  | tmax.dry.mean | | 0.32 |  | -1.81 |  | 2.45 |  |  |
| Strombosia grandifolia | | | |  |  |  |  |  |  |  |
|  |  | intercept |  | -0.22 |  | -1.06 |  | 0.61 |  |  |
|  |  | hunting1 |  | -0.44 |  | -10.50 |  | 9.37 |  |  |
|  |  | logging1 |  | 1.40 |  | -7.17 |  | 10.10 |  |  |
|  |  | Dist |  | 0.13 |  | -0.26 |  | 0.51 |  |  |
|  |  | K |  | 0.04 |  | -0.11 |  | 0.18 |  |  |
|  |  | P |  | 0.43 |  | -0.92 |  | 1.79 |  |  |
|  |  | Ph |  | -2.74 |  | -10.20 |  | 4.63 |  |  |
|  |  | TKN |  | 0.01 |  | 0.00 |  | 0.02 |  |  |
|  |  | tmin.dry.mean | | -0.76 |  | -3.49 |  | 1.97 |  |  |
|  |  | pr.dry.mean |  | -0.03 |  | -0.08 |  | 0.03 |  |  |
|  |  | tmax.dry.mean | | 0.55 |  | -1.50 |  | 2.62 |  |  |
| Strombosia nigropunctata | | | | |  |  |  |  |  |  |
|  |  | intercept |  | 1.77 |  | 0.81 |  | 2.77 |  | * |
|  |  | hunting1 |  | 13.00 |  | 1.89 |  | 24.70 |  | * |
|  |  | logging1 |  | -5.00 |  | -15.00 |  | 5.49 |  |  |
|  |  | Dist |  | 0.53 |  | 0.09 |  | 1.01 |  | * |
|  |  | K |  | -0.22 |  | -0.43 |  | 0.00 |  | * |
|  |  | P |  | 2.18 |  | 0.20 |  | 4.24 |  | * |
|  |  | Ph |  | 14.40 |  | 4.80 |  | 24.20 |  | * |
|  |  | TKN |  | -0.01 |  | -0.03 |  | 0.01 |  |  |
|  |  | tmin.dry.mean | | -1.69 |  | -5.62 |  | 2.26 |  |  |
|  |  | pr.dry.mean |  | 0.01 |  | -0.07 |  | 0.09 |  |  |
|  |  | tmax.dry.mean | | -0.41 |  | -3.43 |  | 2.60 |  |  |
| Strombosia pustulata | | | |  |  |  |  |  |  |  |
|  |  | intercept |  | 0.44 |  | -0.28 |  | 1.16 |  |  |
|  |  | hunting1 |  | 3.74 |  | -5.09 |  | 12.50 |  |  |
|  |  | logging1 |  | 0.07 |  | -8.14 |  | 8.25 |  |  |
|  |  | Dist |  | 0.17 |  | -0.19 |  | 0.52 |  |  |
|  |  | K |  | -0.09 |  | -0.22 |  | 0.05 |  |  |
|  |  | P |  | 0.30 |  | -1.04 |  | 1.64 |  |  |
|  |  | Ph |  | 2.95 |  | -3.57 |  | 9.46 |  |  |
|  |  | TKN |  | 0.00 |  | -0.01 |  | 0.01 |  |  |
|  |  | tmin.dry.mean | | -1.25 |  | -3.85 |  | 1.30 |  |  |
|  |  | pr.dry.mean |  | 0.01 |  | -0.04 |  | 0.06 |  |  |
|  |  | tmax.dry.mean | | 0.70 |  | -1.28 |  | 2.67 |  |  |
| Strombosiopsis tetrandra | | | | |  |  |  |  |  |  |
|  |  | intercept |  | 0.55 |  | -0.16 |  | 1.27 |  |  |
|  |  | hunting1 |  | 4.30 |  | -4.56 |  | 13.20 |  |  |
|  |  | logging1 |  | 0.36 |  | -7.84 |  | 8.53 |  |  |
|  |  | Dist |  | 0.19 |  | -0.16 |  | 0.55 |  |  |
|  |  | K |  | -0.10 |  | -0.24 |  | 0.05 |  |  |
|  |  | P |  | 0.99 |  | -0.35 |  | 2.34 |  |  |
|  |  | Ph |  | 4.93 |  | -1.50 |  | 11.40 |  |  |
|  |  | TKN |  | 0.00 |  | -0.01 |  | 0.01 |  |  |
|  |  | tmin.dry.mean | | 0.73 |  | -1.82 |  | 3.28 |  |  |
|  |  | pr.dry.mean |  | -0.01 |  | -0.06 |  | 0.04 |  |  |
|  |  | tmax.dry.mean | | -1.01 |  | -2.96 |  | 0.92 |  |  |
| Synsepalum longecuneatum | | | | |  |  |  |  |  |  |
|  |  | intercept |  | 0.52 |  | -0.29 |  | 1.36 |  |  |
|  |  | hunting1 |  | 4.88 |  | -4.90 |  | 14.70 |  |  |
|  |  | logging1 |  | -2.49 |  | -11.70 |  | 6.42 |  |  |
|  |  | Dist |  | 0.22 |  | -0.17 |  | 0.61 |  |  |
|  |  | K |  | -0.10 |  | -0.26 |  | 0.06 |  |  |
|  |  | P |  | 1.34 |  | -0.15 |  | 2.87 |  |  |
|  |  | Ph |  | 3.80 |  | -3.37 |  | 11.30 |  |  |
|  |  | TKN |  | 0.00 |  | -0.02 |  | 0.01 |  |  |
|  |  | tmin.dry.mean | | 0.16 |  | -2.44 |  | 2.79 |  |  |
|  |  | pr.dry.mean |  | 0.01 |  | -0.05 |  | 0.06 |  |  |
|  |  | tmax.dry.mean | | -0.77 |  | -2.79 |  | 1.25 |  |  |
| Tabernaemontana penduliflora | | | | |  |  |  |  |  |  |
|  |  | intercept |  | -0.58 |  | -1.48 |  | 0.27 |  |  |
|  |  | hunting1 |  | -3.33 |  | -13.50 |  | 6.63 |  |  |
|  |  | logging1 |  | 2.16 |  | -7.42 |  | 12.00 |  |  |
|  |  | Dist |  | -0.03 |  | -0.43 |  | 0.37 |  |  |
|  |  | K |  | -0.02 |  | -0.20 |  | 0.14 |  |  |
|  |  | P |  | -0.34 |  | -1.87 |  | 1.14 |  |  |
|  |  | Ph |  | -5.79 |  | -13.70 |  | 1.76 |  |  |
|  |  | TKN |  | -0.01 |  | -0.02 |  | 0.01 |  |  |
|  |  | tmin.dry.mean | | 0.04 |  | -2.92 |  | 3.02 |  |  |
|  |  | pr.dry.mean |  | -0.03 |  | -0.09 |  | 0.03 |  |  |
|  |  | tmax.dry.mean | | 1.34 |  | -0.92 |  | 3.62 |  |  |
| Terminalia superba | | |  |  |  |  |  |  |  |  |
|  |  | intercept |  | 0.13 |  | -0.61 |  | 0.89 |  |  |
|  |  | hunting1 |  | -3.34 |  | -12.30 |  | 5.64 |  |  |
|  |  | logging1 |  | -5.56 |  | -13.80 |  | 2.56 |  |  |
|  |  | Dist |  | -0.25 |  | -0.60 |  | 0.11 |  |  |
|  |  | K |  | 0.01 |  | -0.13 |  | 0.14 |  |  |
|  |  | P |  | -0.35 |  | -1.67 |  | 0.96 |  |  |
|  |  | Ph |  | 2.53 |  | -4.06 |  | 9.22 |  |  |
|  |  | TKN |  | -0.01 |  | -0.02 |  | 0.00 |  |  |
|  |  | tmin.dry.mean | | -1.90 |  | -4.63 |  | 0.77 |  |  |
|  |  | pr.dry.mean |  | 0.00 |  | -0.05 |  | 0.06 |  |  |
|  |  | tmax.dry.mean | | 1.67 |  | -0.42 |  | 3.79 |  |  |
| Thomandersia hensii | | | |  |  |  |  |  |  |  |
|  |  | intercept |  | 0.85 |  | 0.02 |  | 1.72 |  | * |
|  |  | hunting1 |  | 3.58 |  | -6.21 |  | 13.40 |  |  |
|  |  | logging1 |  | -4.77 |  | -13.30 |  | 3.65 |  |  |
|  |  | Dist |  | 0.18 |  | -0.21 |  | 0.57 |  |  |
|  |  | K |  | -0.12 |  | -0.28 |  | 0.04 |  |  |
|  |  | P |  | 0.59 |  | -0.81 |  | 2.00 |  |  |
|  |  | Ph |  | 8.40 |  | 0.82 |  | 16.30 |  | * |
|  |  | TKN |  | -0.01 |  | -0.02 |  | 0.01 |  |  |
|  |  | tmin.dry.mean | | -0.79 |  | -3.50 |  | 1.91 |  |  |
|  |  | pr.dry.mean |  | 0.00 |  | -0.06 |  | 0.06 |  |  |
|  |  | tmax.dry.mean | | -0.21 |  | -2.33 |  | 1.90 |  |  |
| Trichilia prieuriana | | |  |  |  |  |  |  |  |  |
|  |  | intercept |  | 0.27 |  | -0.43 |  | 0.98 |  |  |
|  |  | hunting1 |  | -0.31 |  | -8.93 |  | 8.45 |  |  |
|  |  | logging1 |  | 0.15 |  | -7.99 |  | 8.17 |  |  |
|  |  | Dist |  | -0.08 |  | -0.42 |  | 0.27 |  |  |
|  |  | K |  | -0.12 |  | -0.26 |  | 0.01 |  |  |
|  |  | P |  | 0.78 |  | -0.54 |  | 2.10 |  |  |
|  |  | Ph |  | 3.11 |  | -3.24 |  | 9.47 |  |  |
|  |  | TKN |  | 0.01 |  | 0.00 |  | 0.02 |  |  |
|  |  | tmin.dry.mean | | -1.24 |  | -3.74 |  | 1.28 |  |  |
|  |  | pr.dry.mean |  | 0.02 |  | -0.03 |  | 0.07 |  |  |
|  |  | tmax.dry.mean | | 0.75 |  | -1.18 |  | 2.69 |  |  |
| Trichilia rubescens | | |  |  |  |  |  |  |  |  |
|  |  | intercept |  | 0.25 |  | -0.47 |  | 0.97 |  |  |
|  |  | hunting1 |  | -4.01 |  | -12.90 |  | 4.91 |  |  |
|  |  | logging1 |  | -3.45 |  | -11.80 |  | 4.89 |  |  |
|  |  | Dist |  | -0.32 |  | -0.67 |  | 0.03 |  |  |
|  |  | K |  | 0.09 |  | -0.05 |  | 0.23 |  |  |
|  |  | P |  | -0.11 |  | -1.49 |  | 1.31 |  |  |
|  |  | Ph |  | 6.00 |  | -0.39 |  | 12.40 |  |  |
|  |  | TKN |  | 0.02 |  | 0.01 |  | 0.03 |  | * |
|  |  | tmin.dry.mean | | 1.00 |  | -1.53 |  | 3.54 |  |  |
|  |  | pr.dry.mean |  | -0.02 |  | -0.08 |  | 0.03 |  |  |
|  |  | tmax.dry.mean | | -1.51 |  | -3.49 |  | 0.44 |  |  |
| Trichilia welwitschii | | | |  |  |  |  |  |  |  |
|  |  | intercept |  | 0.36 |  | -0.42 |  | 1.17 |  |  |
|  |  | hunting1 |  | 0.48 |  | -8.76 |  | 9.77 |  |  |
|  |  | logging1 |  | -2.38 |  | -10.70 |  | 5.75 |  |  |
|  |  | Dist |  | -0.21 |  | -0.57 |  | 0.14 |  |  |
|  |  | K |  | 0.07 |  | -0.07 |  | 0.20 |  |  |
|  |  | P |  | 0.28 |  | -1.06 |  | 1.61 |  |  |
|  |  | Ph |  | 4.93 |  | -1.98 |  | 12.00 |  |  |
|  |  | TKN |  | 0.00 |  | -0.01 |  | 0.01 |  |  |
|  |  | tmin.dry.mean | | 1.86 |  | -0.85 |  | 4.52 |  |  |
|  |  | pr.dry.mean |  | -0.08 |  | -0.13 |  | -0.02 |  | * |
|  |  | tmax.dry.mean | | -1.41 |  | -3.51 |  | 0.70 |  |  |
| Vitex welwitschii | | |  |  |  |  |  |  |  |  |
|  |  | intercept |  | 0.25 |  | -0.55 |  | 1.04 |  |  |
|  |  | hunting1 |  | 5.25 |  | -5.69 |  | 16.00 |  |  |
|  |  | logging1 |  | -2.97 |  | -13.60 |  | 7.90 |  |  |
|  |  | Dist |  | -0.03 |  | -0.46 |  | 0.39 |  |  |
|  |  | K |  | 0.01 |  | -0.15 |  | 0.16 |  |  |
|  |  | P |  | 0.63 |  | -1.05 |  | 2.32 |  |  |
|  |  | Ph |  | -0.12 |  | -7.09 |  | 6.93 |  |  |
|  |  | TKN |  | -0.01 |  | -0.02 |  | 0.01 |  |  |
|  |  | tmin.dry.mean | | -0.12 |  | -3.13 |  | 2.89 |  |  |
|  |  | pr.dry.mean |  | 0.03 |  | -0.04 |  | 0.09 |  |  |
|  |  | tmax.dry.mean | | -0.12 |  | -2.33 |  | 2.09 |  |  |
| Xylopia chrysophylla | | | |  |  |  |  |  |  |  |
|  |  | intercept |  | 0.11 |  | -0.70 |  | 0.91 |  |  |
|  |  | hunting1 |  | 6.39 |  | -4.30 |  | 17.10 |  |  |
|  |  | logging1 |  | 4.61 |  | -5.77 |  | 16.10 |  |  |
|  |  | Dist |  | 0.27 |  | -0.17 |  | 0.74 |  |  |
|  |  | K |  | 0.02 |  | -0.13 |  | 0.17 |  |  |
|  |  | P |  | -0.65 |  | -2.38 |  | 1.05 |  |  |
|  |  | Ph |  | -2.33 |  | -9.90 |  | 4.92 |  |  |
|  |  | TKN |  | 0.00 |  | -0.02 |  | 0.01 |  |  |
|  |  | tmin.dry.mean | | -0.86 |  | -3.82 |  | 2.01 |  |  |
|  |  | pr.dry.mean |  | 0.04 |  | -0.02 |  | 0.10 |  |  |
|  |  | tmax.dry.mean | | 0.38 |  | -1.76 |  | 2.57 |  |  |
| Xylopia phloiodora | | |  |  |  |  |  |  |  |  |
|  |  | intercept |  | 0.52 |  | -0.26 |  | 1.33 |  |  |
|  |  | hunting1 |  | 5.23 |  | -4.91 |  | 15.60 |  |  |
|  |  | logging1 |  | 0.19 |  | -8.33 |  | 8.89 |  |  |
|  |  | Dist |  | 0.27 |  | -0.13 |  | 0.68 |  |  |
|  |  | K |  | -0.09 |  | -0.25 |  | 0.06 |  |  |
|  |  | P |  | -0.53 |  | -2.08 |  | 1.00 |  |  |
|  |  | Ph |  | 3.40 |  | -3.68 |  | 10.60 |  |  |
|  |  | TKN |  | -0.01 |  | -0.02 |  | 0.01 |  |  |
|  |  | tmin.dry.mean | | -0.49 |  | -3.29 |  | 2.32 |  |  |
|  |  | pr.dry.mean |  | 0.01 |  | -0.05 |  | 0.07 |  |  |
|  |  | tmax.dry.mean | | 0.18 |  | -2.01 |  | 2.38 |  |  |
| Zanthoxylum gilletii | | | |  |  |  |  |  |  |  |
|  |  | intercept |  | 0.00 |  | -0.90 |  | 0.91 |  |  |
|  |  | hunting1 |  | -11.50 |  | -23.30 |  | 0.37 |  |  |
|  |  | logging1 |  | -11.60 |  | -21.80 |  | -1.70 |  | * |
|  |  | Dist |  | -0.75 |  | -1.25 |  | -0.24 |  | * |
|  |  | K |  | -0.09 |  | -0.27 |  | 0.08 |  |  |
|  |  | P |  | -0.14 |  | -1.76 |  | 1.47 |  |  |
|  |  | Ph |  | 6.45 |  | -1.05 |  | 14.00 |  |  |
|  |  | TKN |  | -0.01 |  | -0.03 |  | 0.00 |  | * |
|  |  | tmin.dry.mean | | 1.67 |  | -2.24 |  | 5.62 |  |  |
|  |  | pr.dry.mean |  | 0.00 |  | -0.08 |  | 0.07 |  |  |
|  |  | tmax.dry.mean | | -0.15 |  | -2.92 |  | 2.66 |  |  |

## A4: Table of community sensitivity to environmental covariates. Sensitivity is dimensionless and on the scale of the predictor.

| Community Sensitivities to Predictors | | | | | | |
| --- | --- | --- | --- | --- | --- | --- |
|  |  |  |  |  |  |  |
| Predictor |  | Estimate |  | 2.5% |  | 97.5% |
| Dry Season Precip. | | 0.916 |  | 0.756 |  | 1.09 |
| Soil pH |  | 1.02 |  | 0.847 |  | 1.2 |
| Soil K |  | 1.08 |  | 0.908 |  | 1.27 |
| Soil P |  | 1.11 |  | 0.922 |  | 1.31 |
| Soil TKN |  | 1.15 |  | 0.94 |  | 1.38 |
| Dry Season Max. Temp. | | 1.28 |  | 1.06 |  | 1.51 |
| Dry Season Min. Temp. | | 1.48 |  | 1.21 |  | 1.78 |
| Logging |  | 1.99 |  | 1.65 |  | 2.35 |
| Hunting |  | 2.22 |  | 1.85 |  | 2.61 |
| Distance to Village | | 3.29 |  | 2.76 |  | 3.85 |

## A5: Trait-specific posterior parameter estimates for the effect of environmental covariates on community weighted trait values.

## A6: Table of community weighted trait sensitivity to environmental covariates. Sensitivity is dimensionless and on the scale of the predictor.

## A7: Community weighted trait ordination suggests that species are clustered into pioneer and secondary communities.

##
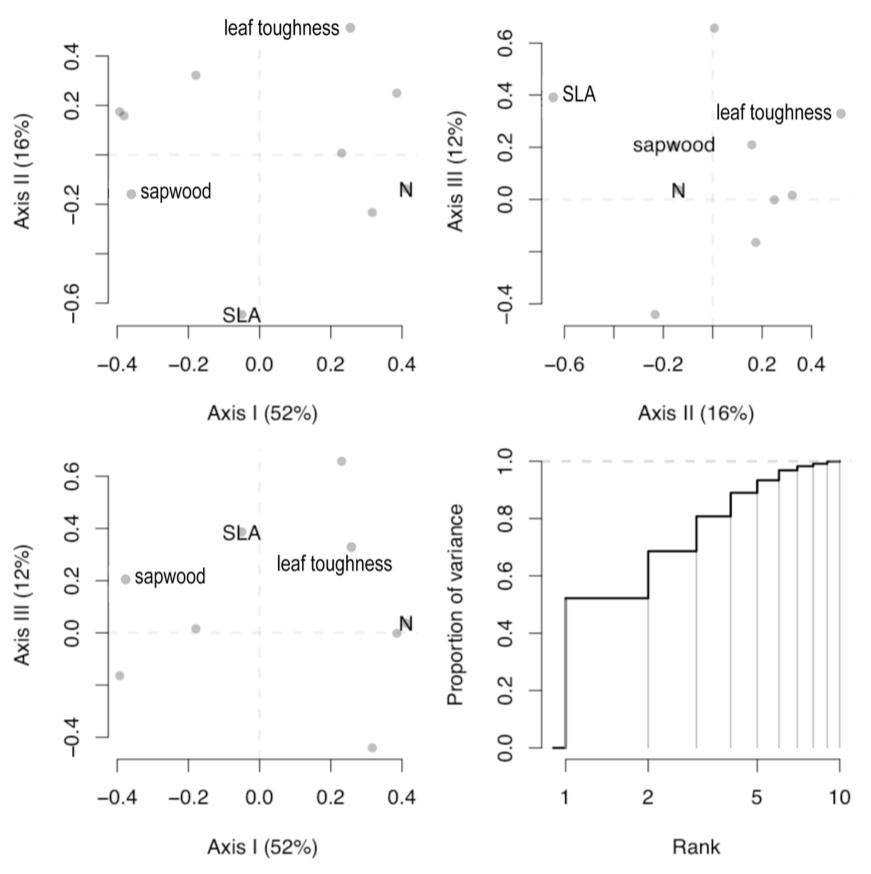


## A8: Dendrogram demonstrating clustering by correlation in trait data (A) and correlation by trait response to environment (B).

## A9: Ordered species names from figure 2 are clustered into a disturbance tolerant group (1), and a disturbance intolerant group (2).

##
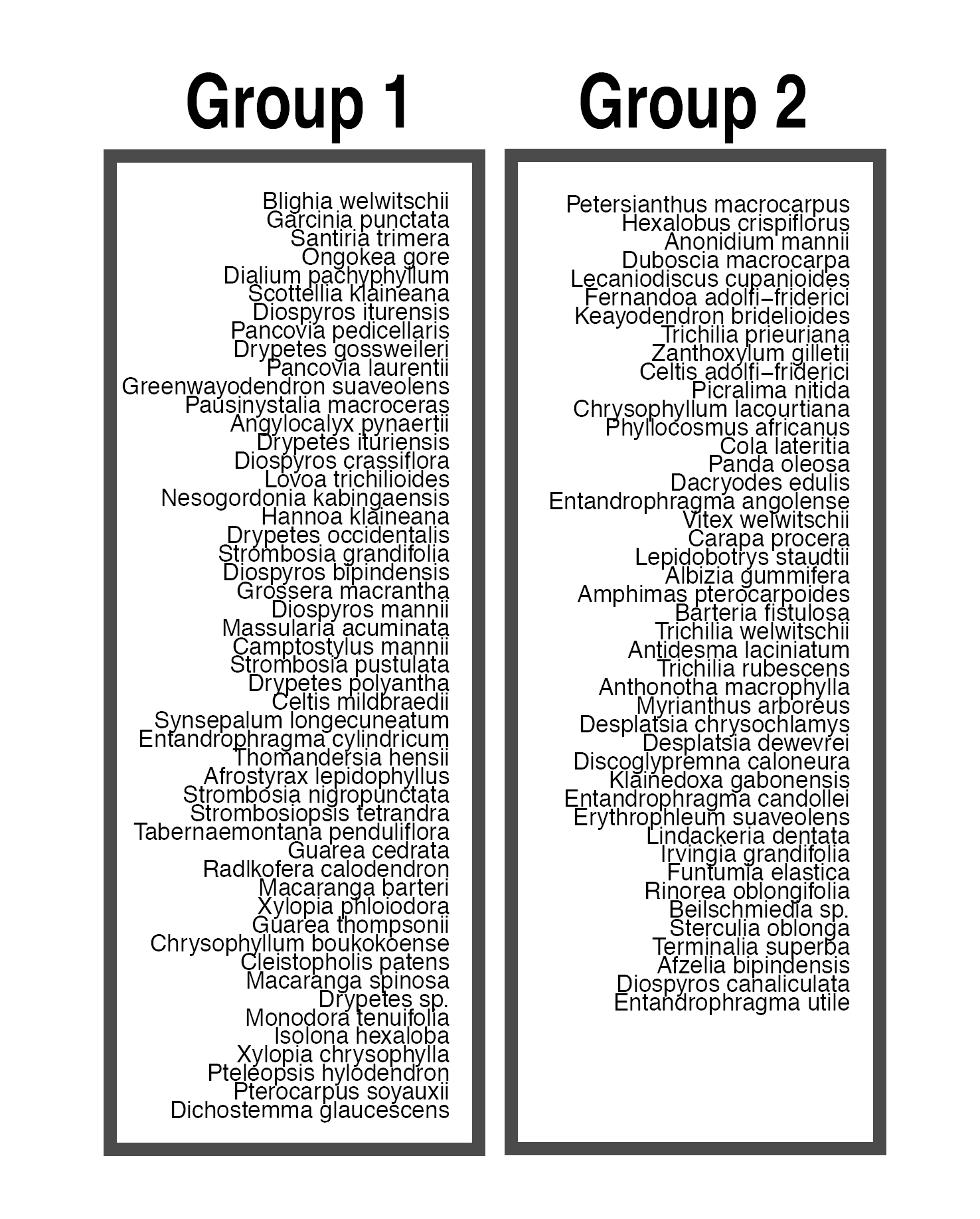

Supplement: Supplementary file 1 — Appendix S1 [file ECE3-14-e11329-s001.docx]
